# Filamin A editing in myeloid cells reduces intestinal inflammation and protects from colitis

**DOI:** 10.1084/jem.20240109

**Published:** 2025-06-05

**Authors:** Riem Gawish, Rajagopal Varada, Florian Deckert, Anastasiya Hladik, Linda Steinbichl, Laura Cimatti, Katarina Milanovic, Mamta Jain, Natalya Torgasheva, Andrea Tanzer, Kim De Paepe, Tom Van de Wiele, Bela Hausmann, Michaela Lang, Martin Pechhacker, Nahla Ibrahim, Ingrid De Vries, Christine Brostjan, Michael Sixt, Christoph Gasche, Louis Boon, David Berry, Michael F. Jantsch, Fatima C. Pereira, Cornelia Vesely

**Affiliations:** 1 Center of Anatomy and Cell Biology, Division of Cell and Developmental Biology, Medical University of Vienna, Vienna, Austria; 2 Department of Medicine I, Research Division of Infection Biology, Medical University of Vienna, Vienna, Austria; 3 Department of Microbiology and Ecosystem Science, University of Vienna, Center for Microbiology and Environmental Systems Science, Vienna, Austria; 4 Department of Biotechnology, Ghent University, Center for Microbial Ecology and Technology, Ghent, Belgium; 5 Joint Microbiome Facility of the Medical University of Vienna and the University of Vienna, Vienna, Austria; 6 Division of Clinical Microbiology, Department of Laboratory Medicine, Medical University of Vienna, Vienna, Austria; 7 Division of Vascular Surgery, Department of General Surgery, Medical University of Vienna, Vienna, Austria; 8 Institute of Science and Technology Austria, Klosterneuburg, Austria; 9 Department of Medicine III, Division of Gastroenterology and Hepatology, Medical University of Vienna, Vienna, Austria; 10 JJP Biologics, Warsaw, Poland; 11 School of Biological Sciences, Faculty for Environmental and Life Sciences, University of Southampton, Southampton, UK

## Abstract

Patho-mechanistic origins of ulcerative colitis are still poorly understood. The actin cross-linker filamin A (FLNA) impacts cellular responses through interaction with cytosolic proteins. Posttranscriptional A-to-I editing generates two forms of FLNA: genome-encoded FLNA^Q^ and FLNA^R^. FLNA is edited in colon fibroblasts, smooth muscle cells, and endothelial cells. We found that the FLNA editing status determines colitis severity. Editing was highest in healthy colons and reduced during murine and human colitis. Mice that exclusively express FLNA^R^ were highly resistant to DSS-induced colitis, whereas fully FLNA^Q^ animals developed severe inflammation. While the genetic induction of FLNA editing influenced transcriptional states of structural cells and microbiome composition, we found that FLNA^R^ exerts protection specifically via myeloid cells, which are physiologically unedited. Introducing fixed FLNA^R^ did not hamper cell migration but reduced macrophage inflammation and rendered neutrophils less prone to NETosis. Thus, loss of FLNA editing correlates with colitis severity, and targeted editing of myeloid cells serves as a novel therapeutic approach in intestinal inflammation.

## Teaser

In this study, Gawish et al. show that RNA editing of the actin cross-linker FLNA is similarly regulated in mice and humans and that the targeted induction of edited FLNAR in myeloid cells governs resistance to DSS-induced colitis, revealing its potential in IBD therapy.

## Introduction

The actin cytoskeleton is a complex and dynamic network composed of actin filaments and binding proteins, which define cell morphology and function. Filamin A (FLNA) is a large actin–cross-linking protein, ubiquitously expressed and composed of 24 Ig-like domains. Functionally, FLNA links actin filaments with the cellular cortex and, via Ig-domains 21–23, acts as a scaffolding platform for a variety of proteins, thereby tuning cellular signaling (Nakamura et al., 2011). FLNA deficiency in mice is embryonically lethal, causing cardiac defects and abnormal epithelial and endothelial organization (Feng et al., 2006). In humans, *FLNA* mutations cause a broad range of congenital disorders, highlighting its cell type–specific functions (Wade et al., 2020). Interestingly, certain FLNA mutations cause gastrointestinal dysfunction like severe constipation, intestinal pseudo-obstruction, and short bowel syndrome. Likewise, FLNA critically influences intestinal development in mice (Gargiulo et al., 2007). FLNA mRNA is posttranscriptionally modified by adenosine (A) to inosine (I) editing, an RNA modification catalyzed by the adenosine deaminase acting on double-stranded RNA 2 (ADAR2). As I is interpreted as guanosine (G) by most cellular machineries, exonic A-to-I editing can cause protein recoding and proteome diversification. Aside from the physiological functions of A-to-I editing, genetically engineered ADAR enzymes can be redirected to specific targets. After the first demonstration of site-directed RNA editing (SDRE), the potential for therapeutic RNA editing became more and more evident, with recent approaches mainly focusing on the transient and cell modulatory features of SDRE (Khosravi and Jantsch, 2021; Diaz Quiroz et al., 2023).

FLNA recoding is highly conserved in vertebrates and changes a glutamate (Q) to an arginine (R) right in the center of the protein interaction–scaffolding region of FLNA (Ig-domain 22) (Stulić and Jantsch, 2013). Using mice that exclusively express unedited FLNA (FLNA^Q^) or edited FLNA (FLNA^R^) (Jain et al., 2018, 2022a), we recently showed that FLNA^R^ increases cellular stiffness and adhesion in murine fibroblasts, while FLNA^Q^ renders cells more flexible and facilitates cell migration (Jain et al., 2022b). In mice, FLNA is edited in vasculature and, accordingly, regulates blood pressure (Jain et al., 2018) and promotes tumor angiogenesis (Jain et al., 2022a). In the murine gut, FLNA editing is high, with 80–90% in the stomach and 60–80% in the large intestine (Stulić and Jantsch, 2013; Szabo et al., 2024). In human colon biopsies, editing frequencies up to 26% have been reported (Gabay et al., 2022). However, the role of FLNA editing in the gut is still unexplored as of today.

The gastrointestinal tract requires a tight regulation of immune responses and epithelial cell (EC) regeneration to combat environmental threats without the development of pathological inflammation. This is achieved by a highly adapted immune compartment, which is critically shaped by constant cross talk with the microbiome, the key component of a balanced intestinal milieu (Caruso et al., 2020). Ulcerative colitis (UC) is a chronic or remitting inflammatory bowel disease (IBD) of the colonic mucosa affecting up to 0.42% of individuals in industrialized countries (National Library of Medicine, 2023). Intestinal inflammation in UC patients typically spreads from the distal colorectum toward the proximal parts of the colon. Risk alleles for UC include genes important for epithelial barrier integrity or immune regulation but explain only 7.5% of disease variants, while environmental factors and dysbiosis shape disease onset and severity (Jostins et al., 2012). In fact, the pathophysiology of UC is driven by a triad of dysbiosis, barrier dysfunction, and immune activation, but the actual sequence of patho-mechanistic events remains incompletely understood. In mice, UC can be mimicked by supplementation of the drinking water with dextran sodium sulfate (DSS). DSS disrupts the barrier layer, followed by entry of commensal bacteria into the mucosa and subsequent immune activation (Eichele and Kharbanda, 2017). Importantly, the DSS sensitivity of mice highly depends on their hygiene status, again highlighting microbiome and host immune factors as critical determinants of disease severity (Brinkman et al., 2013). In addition, EC survival and regeneration are key parameters for barrier integrity, which ultimately reduces inflammation (Thoo et al., 2019).

Based on the elevated FLNA editing in the colon (Stulić and Jantsch, 2013) and the fact that specific mutations result in intestinal dysfunction (Gargiulo et al., 2007), we hypothesized that the FLNA editing state may, similar to certain point mutations, impact intestinal development and/or immune homeostasis, and we specifically investigated the impact of genetically fixed FLNA editing states by assessing immunity, barrier function, and microbiome composition in naïve and DSS-challenged FLNA mutant mice.

## Results

### FLNA editing impacts cell transcriptional profiles and inflammation in the healthy gut

**Figure 1.**
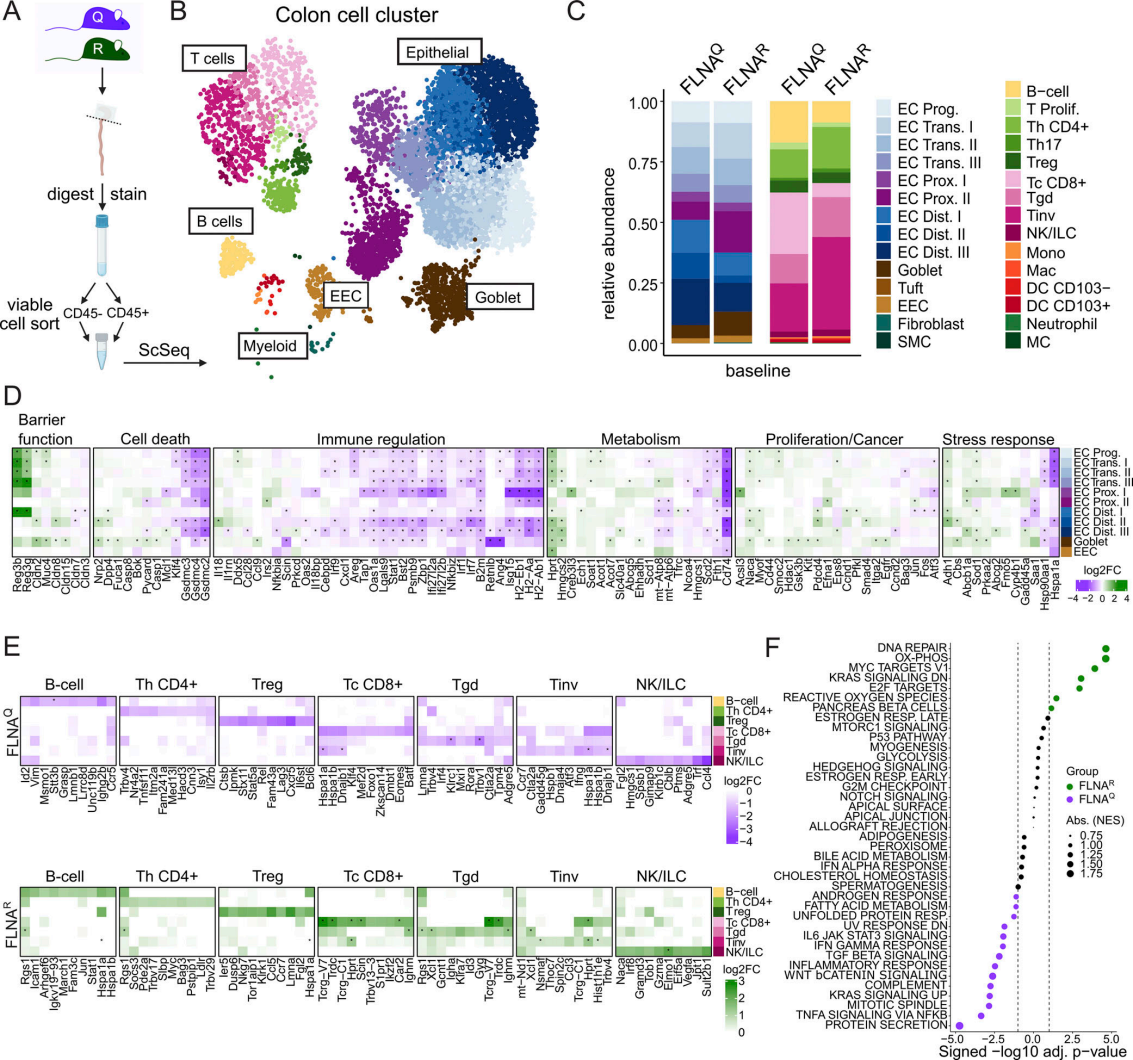
**FLNA editing impacts cell transcriptional profiles and inflammation in the healthy gut. (A)** Scheme of scRNA-seq setup for FLNA^R^ and FLNA^Q^ mouse colons at basal conditions. **(B)** Cell clustering of colon mucosal cells. **(C)** Relative abundance of parenchymal (CD45^−^) and immune (CD45^+^) cells in FLNA^R^ and FLNA^Q^ intestines; left panel: EC progenitors (EC Prog.), ECs trans (I–III), proximal ECs (EC Prox. I–II), distal ECs (EC Dist. I–III), goblet cells, tuft cells, enteroendocrine cells (EEC), fibroblasts, and SMCs; right panel: B cells, proliferating T cells (T Prolif.), T helper CD4^+^ (Th CD4^+^), T helper 17 cells (Th17), regulatory T cells (Treg), classical cytotoxic CD8^+^ T cells (Tc CD8^+^), γδT cells (Tgd), invariant T cells (Tinv), NK/innate lymphoid cells (ILC), monocytes (Mono), macrophages (Mac), CD103^−^ and CD103^+^ DCs, neutrophils, and mast cells (MC). **(D)** Heatmaps of transcriptomic analysis of DEGs in intestinal structural cells of FLNA^R^ and FLNA^Q^ mice grouped by cellular processes. Heatmap color represents the log_2_-fold increase of expression in FLNA^R^ (green) versus FLNA^Q^ (purple). Rows represent specific cell types. Asterisks represent adjusted values of P < 0.05. **(E)** Heatmaps of transcriptomic analysis of DEGs in intestinal immune cells of FLNA^R^ and FLNA^Q^ mice grouped by cell type. Heatmap color represents log_2_-fold change of expression in FLNA^R^ (green) and FLNA^Q^ (purple). Rows represent specific cell types. Asterisks represent adjusted values of P < 0.05. **(F)** Bubble plot representing top 40 MSigDB HALLMARK pathways enriched in bulk RNA sequencing transcriptomes of FLNA^R^ (green) and FLNA^Q^ (purple) cells. Circle sizes indicate the number of DEGs associated with the respective pathway. Data in A–F are from single experiments (three mice pooled per group for single-cell sequencing, *n* = 4–5 per group for bulk sequencing). NES, normalized enrichment score.

The *Flna* mRNA is reported to be edited 60–80% in the healthy murine colon (Stulić and Jantsch, 2013; Szabo et al., 2024). Using FLNA mutant mice that exclusively express either FLNA^R^ or FLNA^Q^ (Jain et al., 2018, 2022a), we first asked how the FLNA editing state impacts cellular transcriptomes under homeostatic conditions. We generated single-cell suspensions derived from the epithelial layer (containing intraepithelial lymphocytes and enterocytes) combined with the lamina propria immune cell fraction from healthy FLNA^R^ and FLNA^Q^ colons and sorted viable CD45^−^ and CD45^+^ cells. Upon single-cell sequencing (Fig. 1 A), we identified nine clusters of EC states, including progenitors, transitional ECs (EC trans I–III), distal ECs (Dist I–III), and proximal ECs (Prox I–II), as well as goblet cells, enteroendocrine cells, and a few fibroblasts, tuft cells, and smooth muscle cells (SMCs) (Fig. 1 B). The immune cell compartment consisted of different lymphocyte subsets, including classical CD4^+^ T helper cells (Th cells), regulatory T and Th17 helper cells, three subtypes of CD8^+^ T cells, a small cluster of innate lymphoid cells, and B cells (Fig. 1 B). Myeloid cells were sparse, consisting of macrophages, monocytes, dendritic cells (DCs), and a few neutrophils and mast cells (Fig. 1 B).

**Figure S1.**
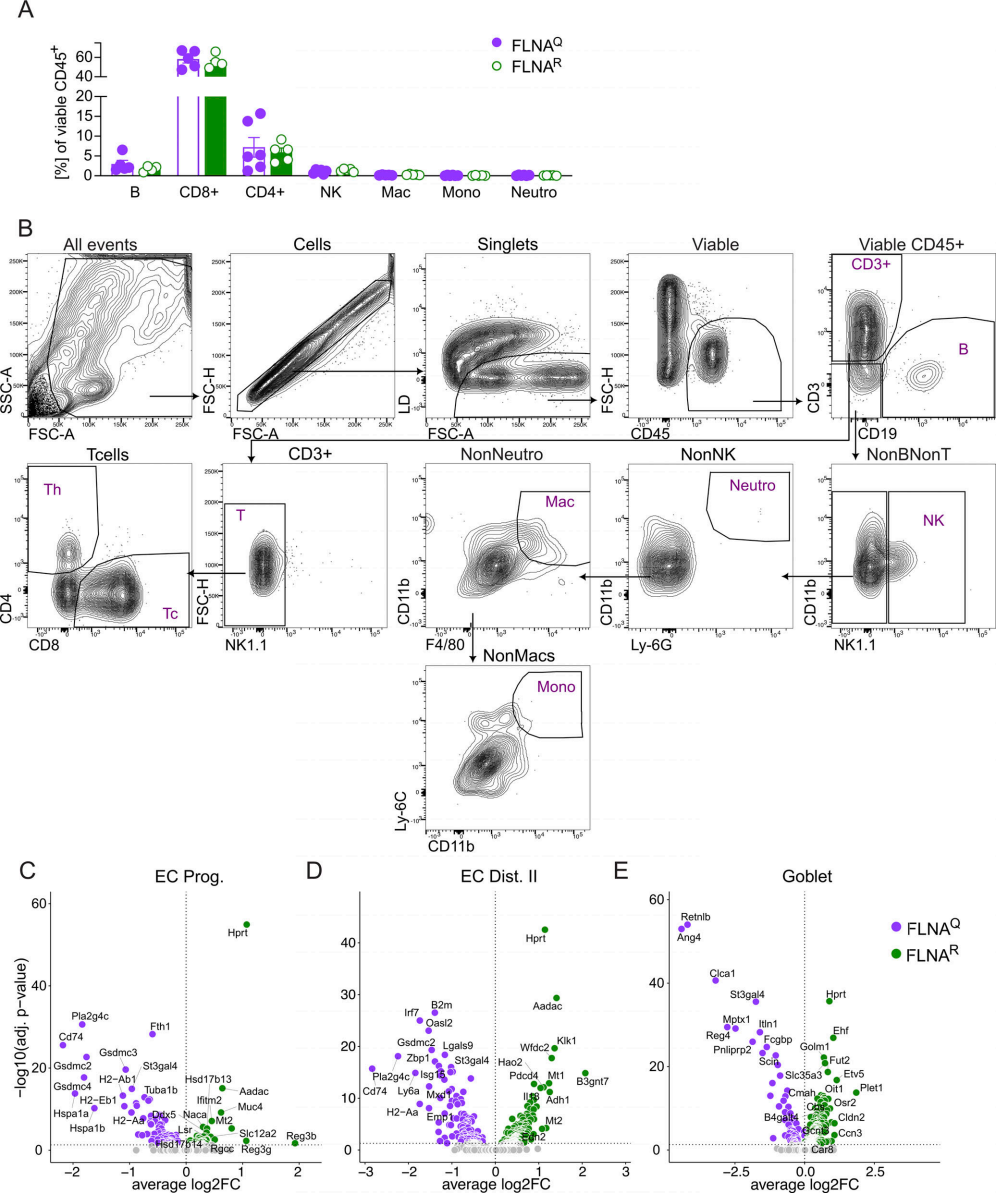
**FLNA editing impacts epithelial transcriptional profiles and inflammation in the healthy gut. (A)** Relative numbers of immune cells in healthy mouse colons (Student’s *t* test). **(B–E)** Gating strategy of colon tissue FACS analysis. Volcano plots (DEGs, Padj ≤ 0.05) of FLNA^R^ versus FLNA^Q^ cells identified by DESeq2 analysis for (C) EC progenitors, (D) distal EC type II, and (E) goblet cells. Data in A and B are representative of two independent experiments; data in C–E are from single experiments (three mice pooled per group for single-cell sequencing). Mono, monocyte; Mac, macrophages; Neutro, neutrophil.

Among structural cells, compositional analysis suggested that FLNA^R^ colons harbored more ECs trans, proximal ECs, and goblet cells but less differentiated distal ECs than FLNA^Q^ colons (Fig. 1 C, left panel). Moreover, we isolated more atypical CD8^+^ T (γδT cells and invariant T cells) and CD4^+^ Th cells from FLNA^R^ colons, at the cost of a slightly smaller B and classical CD8^+^ T cell fraction (Fig. 1 C, right panel). However, flow cytometric analysis of CD45^+^ cell numbers did not reveal significant differences between groups with regard to CD8^+^ T cells (covering classical and atypical CD8^+^ T cells), CD4^+^ T cells, B cells, or myeloid cells between the groups (Fig. S1, A and B). Differentially expressed genes (DEGs) were determined for populations with at least 10 cells per genotype, hence excluding fibroblasts, SMCs, tuft cells, T helper 17 cells, and all myeloid cells from analysis (Table S1). Profound differences were found in the EC compartment, namely in progenitors, early EC trans, distal EC, and goblet cells (Fig. S1, C–E). Interestingly, genes associated with epithelial barrier function (*Reg3b/g*, *Cldn2/8/15*, and *Muc4*) (Shindo et al., 2020; Cornick et al., 2015; Čužić et al., 2021) were upregulated in FLNA^R^ ECs, while FLNA^Q^ promoted a proinflammatory profile, with higher expression of genes involved in antigen presentation (e.g., *H2-Aa/Ab1/Eb1* and *B2m* [Jhunjhunwala et al., 2021]), IFN signaling (e.g., *Isg15*, *Stat1*, *Irf1/7/9*, *Bst2*, *Oas1a/2*, and *Tap1*) (Schneider et al., 2014), and neutrophil recruitment (*Cxcl1*) (Sawant et al., 2016) (Fig. 1 D, Fig. S1, C–E, and Table S2). ECs also showed expression of genes involved in cell death. Gasdermins (*Gsdmc2/3/4*), caspase 1 (*casp1*), Asc (*pycard*), important pyroptosis-related genes (Fang et al., 2022), and factors involved in ferroptosis, another type of inflammatory cell death, were highly expressed in FLNA^Q^ ECs (e.g., *Ncoa4* and *Fth1*) (Tang et al., 2021). Further, the FLNA editing state influenced genes associated with proliferation, cancer, and stress response, with some being upregulated in FLNA^R^ (e.g., *Naca*, *Myof*, *Smoc2*, and *Hprt*) (Gong et al., 2010; Dong et al., 2019; Su et al., 2016; Wang et al., 2021) and others being upregulated in FLNA^Q^ ECs, like the AP-1 transcription factor complex (*Atf3*, *Fos*, and *Jun*) (Wu et al., 2021) (Fig. 1 D, Fig. S1, B–D, and Table S2). In contrast to structural cells, differences in immune cells were restricted to a few DEGs found in CD8^+^ T cells associated with inflammation and activation (*Tcrg*, *Trdc*, *Dnajb1*, and *Hspa1a/1b*). Similar to ECs, we found *Hprt* upregulated in FLNA^R^ CD8^+^ T cells, while FLNA^Q^ immune cells expressed more *Fth1*, the ferritin heavy chain, suggesting a cell type–independent impact of FLNA editing on *Hprt* and *Fth1* expression (Fig. 1 E and Table S2). Bulk sequencing from distal colon tissues confirmed *Hprt* upregulation in FLNA^R^ intestines (Table S3), and gene set enrichment analysis (Korotkevich et al., 2021, *Preprint*) confirmed a transcriptional signature that links FLNA^R^ with cell states found in proliferative disorders (DNA repair and Myc signaling) while suppressing inflammation (Fig. 1 F).

Thus, the FLNA editing state impacts gene expression in intestinal parenchymal and immune cells. In ECs, FLNA^R^ promotes a transcriptome associated with proliferative cell states, while FLNA^Q^ drives an inflammatory signature that may promote pyroptotic cell death.

### FLNA^R^ is associated with an immune regulatory gut microbiome

**Figure 2.**
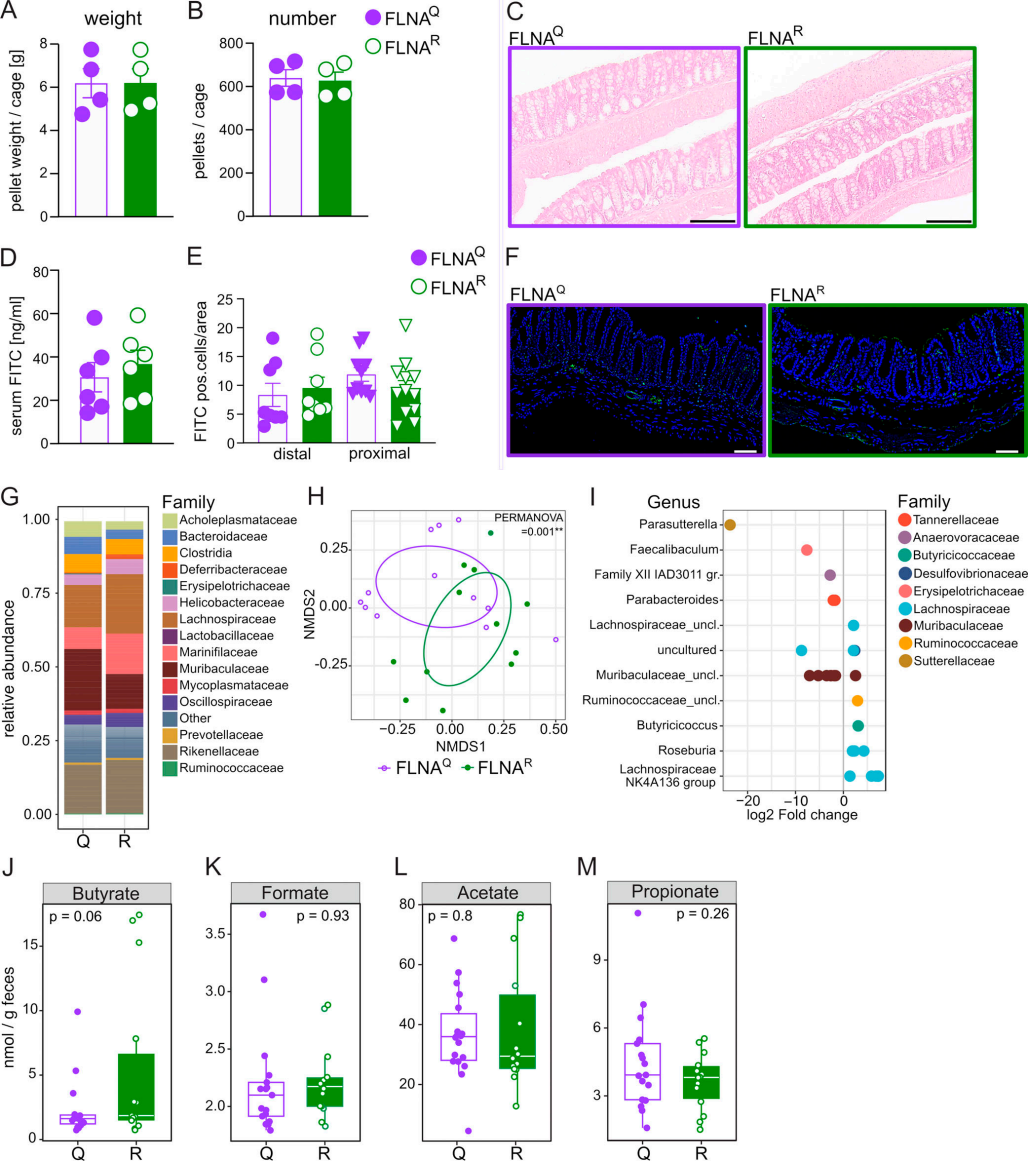
**FLNA** ^**R**^ **is associated with an immune regulatory gut microbiome. (A and B)** (A) Overnight fecal pellet output (weight) and (B) average pellet number per cage of FLNA^Q^ and FLNA^R^ mice (*n* = 16 per genotype, Student’s *t* test). **(C)** Representative H&E-stained colon sections from FLNA^Q^ and FLNA^R^ mice, scale bar = 100 µm. **(D)** Serum fluorescence measurements after oral FITC-dextran gavage calculated as serum FITC in ng/ml (*n* = 6, Student’s *t* test). **(E)** Number of FITC-positive cells/area in cross-sectioned colon tissue after ex vivo incubation of colons with FITC-dextran. Two proximal and two distal colon segments per mouse were analyzed (*n* = 4, Student’s *t* test). **(F)** Representative images of colon cross sections of the distal colon. FITC^+^ cells in green, blue = DAPI, scale bar = 100 µm. **(G)** Family level relative abundance profiles of FLNA^R^ and FLNA^Q^ mice gut microbiomes. Families with relative abundances <2% are collapsed into the category “Other.” Each bar represents the average of multiple samples (*n* = 6 per genotype). **(H)** Ordination plot based on nonmetric multidimensional scaling analysis (NMDS) of Bray–Curtis distances of microbiome profiles at the ASV level. SD ellipses and point dots representing each sample are depicted and are colored by mouse genotype. Stress = 0.114. **(I)** Differential abundant ASVs based on DESeq2 analysis (adjusted value of P < 0.05, Wald test followed by Benjamini–Hochberg correction for multiple testing) between FLNA^R^ and FLNA^Q^ samples (log_2_-fold change >2 denotes enrichment in FLNA^R^ mice; log_2_-fold change less than −2 denotes enrichment in FLNA^Q^ mice). ASVs are assigned to genus (y axis) and colored by family. **(J)** Relative abundance of the genus *Lachnospiraceae* NK4A136 group (left) and ASV_1lf_am9 (right) in fecal samples of FLNA^R^ (R) and FLNA^Q^ (Q) mice. **(K–M)** Concentration of SCFAs butyrate, formate, acetate, and propionate in fecal samples of FLNA^R^ and FLNA^Q^ mice measured by LC-MS (Student’s *t* test). Error bars denote SD. Boxes represent the median, first and third quartiles. Whiskers extend to the highest and lowest values that are within 1.5 times the interquartile range. Data in A and B are derived from four separate cages (four animals per cage, *n* = 16 per genotype). Data in C–N are one experiment.

Despite obvious differences in transcriptional states of intestinal cells, FLNA^R^ and FLNA^Q^ mutants appeared healthy and did not show signs of spontaneous colitis (Jain et al., 2022a). Their bowel movements were normal, indicated by an undistinguishable fecal pellet output in FLNA^Q^ and FLNA^R^ mice (Fig. 2, A and B) and no histological abnormalities (Fig. 2 C). The inflammatory state of intestinal cells depends on the integrity of the epithelial barrier, which shields the deeper immune cell–rich tissue layers from the microbiota but allows small metabolites to diffuse (Caruso et al., 2020). However, we found no indication of barrier dysfunction in naïve animals. Comparable levels of FITC in the serum of both genotypes after oral gavage of FITC-dextran (Fig. 2 D) suggested a similar wall permeability. This was confirmed by ex vivo incubation of colons with FITC-dextran and quantification of the number of FITC^+^ cells present below the EC layer (Fig. 2, E and F).

The intestinal epithelium and its immune system are strongly affected by constant interaction with the microenvironment, while the microbiome is reciprocally shaped by immune factors (Zheng et al., 2020). Considering that, in spite of an intact barrier, inflammatory signatures of intestinal ECs were reduced in FLNA^R^ animals, we hypothesized that FLNA mutant mice might harbor different microbiomes. We thus analyzed fecal pellets from FLNA^R^ and FLNA^Q^ mice, caged according to their genotypes after weaning, by 16S ribosomal RNA sequencing (Fig. 2 G). Indeed, the FLNA editing status influenced the microbiome, as its composition significantly clustered by genotype (Fig. 2 H). While both FLNA^Q^ and FLNA^R^ microbiomes were dominated by typical murine commensals (Fig. 2, G, I, and J), fully FLNA^R^ mice showed a higher abundance of *Lachnospiraceae* and reduced *Parasutterella* and *Muribaculaceae* (Fig. 2, I and J)*.* As *Lachnospiraceae* are known producers of short-chain fatty acids (SCFAs) with important immunomodulatory properties (Ranjbar et al., 2021; Abdugheni et al., 2022), we measured SCFAs and indeed found higher butyrate levels in the feces of FLNA^R^ mice (Fig. 2 K), while propionate, acetate, and formate were comparable between genotypes (Fig. 2, L–N).

Taken together, the FLNA editing state did not impact epithelial barrier integrity but was associated with a potentially more immunomodulatory microbiome in FLNA^R^ mice, which associates with the differential, less inflammatory transcriptional signatures we have observed in FLNA^R^ guts.

### FLNA editing protects mice from DSS-induced colitis

**Figure S2.**
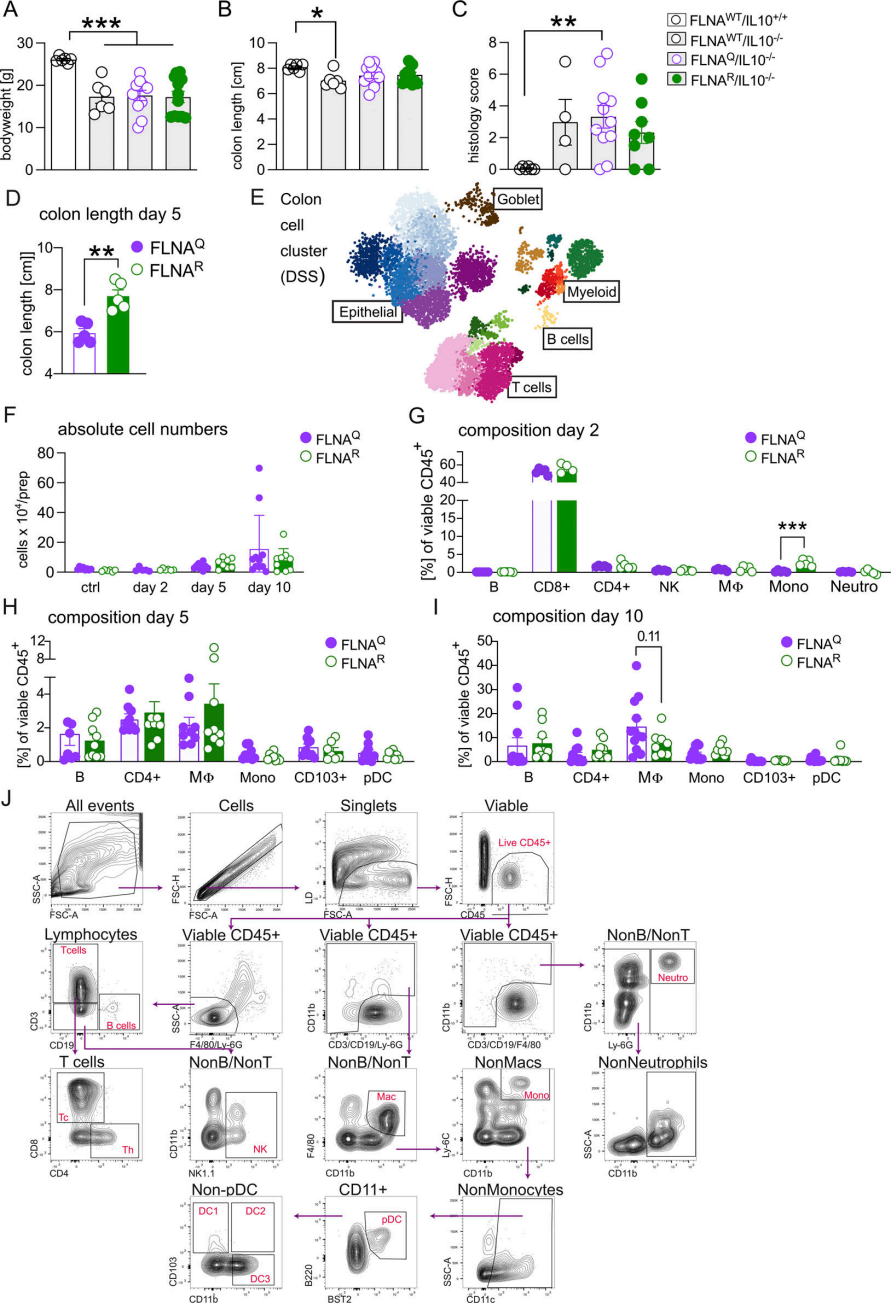
**FLNA editing protects mice from DSS-induced colitis and reduces early inflammatory cytokine production and intestinal neutrophil accumulation. (A)** Body weight of FLNA^WT^/IL-10^+/+^, FLNA^WT^/IL-10^−/−^, FLNA^Q^/IL-10^−/−^, and FLNA^R^/IL-10^−/−^ mice at the time of sacrifice (7–10 wk of age, at onset of symptoms, one-way ANOVA). **(B)** Length measurements of colons at time of sacrifice (one-way ANOVA). **(C)** Total colitis histology score. Higher score means more severe colitis. (Kruskal–Wallis test, FLNA^WT^–IL-10^+/+^, and FLNA^WT^–IL-10^−/−^, *n* = 6; FLNA^Q^–IL-10^−/−^, *n* = 11; FLNA^R^–IL-10^−/−^, *n* = 12). **(D)** Colon length measurements after 5 days of DSS of FLNA^Q^ and FLNA^R^ mice (*n =* 5 for each genotype, Student’s *t* test). **(E)** Cell clustering of large intestine mucosal cells after 5 days of DSS. **(F)** Absolute cell counts of immune cells from colons of mice treated with DSS for 0, 2, 5, and 10 days. **(G–I)** Relative abundance (% of viable CD45^+^ cells) of depicted immune cells of FLNA^R^ and FLNA^Q^ mouse colons at days 2, 5, and 10 of DSS treatment (one-way ANOVA). pDC=plasmacytoid dendritic cells, MΦ=macrophages. **(J)** Gating strategy for flow cytometry of intestinal cells of FLNA^R^ and FLNA^Q^ mice. ***P < 0.001; **P < 0.01; *P < 0.05. Error bars show SEM. Data in A–C and E are from a single experiment. Data in D and F–J are representative of two independent experiments.

Considering the differences in transcriptional cell states and microbiomes, we next sought to test the impact of FLNA editing states on the susceptibility to colitis in an IL-10–deficient background. IL-10^−/−^ animals have an intact epithelial barrier but progressively develop spontaneous colitis due to hyperinflammatory responses of macrophages to the microbiota, which are not counteracted by Treg-derived IL-10 (Kiesler et al., 2015). Fixed FLNA editing states did not significantly impact spontaneous colitis upon IL-10 deficiency. By 8 wk of age, all IL-10^−/−^ animals already showed significant growth retardation and shortened colons independent of the FLNA genotype compared with WT animals (Fig. S2, A and B). No significant difference was seen upon severity scoring of intestinal tissues between FLNA^Q^ IL-10^−/−^ and FLNA^R^ IL-10^−/−^ mice; only FLNA^Q^ IL-10^−/−^ mice scored significantly worse than healthy controls (Fig. S2 C).

We next asked if a more severe disruption of intestinal homeostasis would unveil differences between FLNA mutants using the widely used DSS-induced colitis model (Eichele and Kharbanda, 2017).

**Figure 3.**
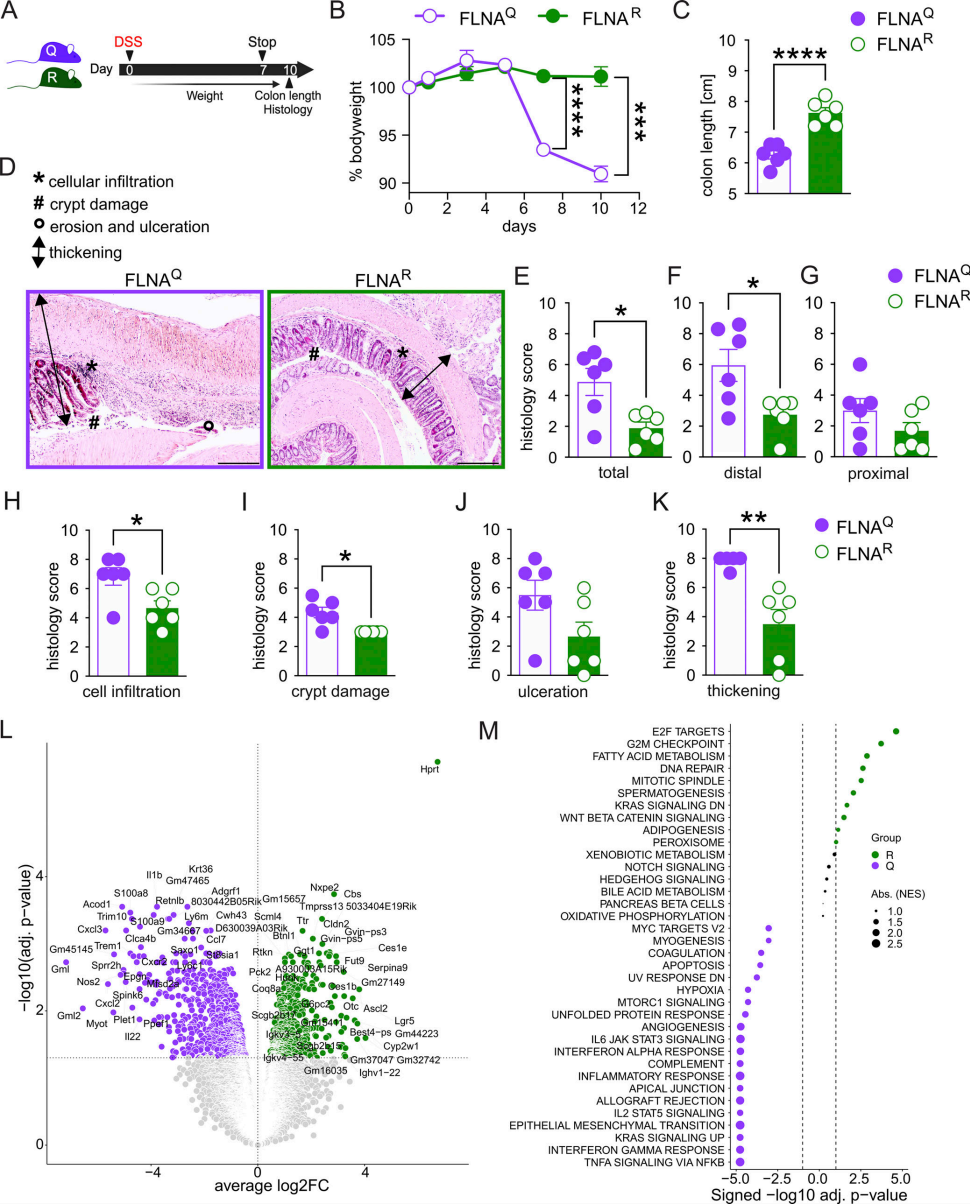
**FLNA editing protects mice from DSS-induced colitis. (A)** Scheme of DSS-induced colitis experiment. **(B)** Weight loss plotted in percent of body weight compared with treatment start (two-way ANOVA, *F* = 45.61, *DF* = 5). **(C)** Length measurements of colons at day 10 (*n* = 6, Student’s *t* test). **(D)** Representative images of histologic colon sections; bar = 100 µm. **(E–G)** (E) Total, (F) distal colon, and (G) proximal colon colitis histology score. A higher score means more severe colitis. (*n* = 6, Mann–Whitney *U* test). **(H–K)** Individual colitis histology scoring parameters for the whole colon length: Cellular (immune cell) infiltration, crypt damage, erosion and ulceration of epithelium, and thickening of colon wall (*n* = 6; Student’s *t* test). **(L)** Volcano plot (DEGs, Padj ≤ 0.05) of FLNA^R^ versus FLNA^Q^ cells identified by DESeq2 analysis in whole distal colon bulk RNA sequencing. **(M)** Bubble plot representing the top 40 MSigDB HALLMARK pathways enriched in bulk transcriptomes of FLNA^R^ (green) and FLNA^Q^ (purple) mice at day 10 of DSS challenge. Circle sizes indicate the number of DEGs associated with the respective pathway. ****P < 0.0001; ***P < 0.001; **P < 0.01; *P < 0.05. Error bars show SEM. Data in A–K are representative of at least three individual experiments. Data in L and M: Bulk RNA sequencing were performed from one experiment (*n* = 4–5 per group for bulk sequencing).

We treated FLNA^R^ and FLNA^Q^ mice with 2–2.5% of DSS in the drinking water for 7 days, followed by 3 days of regular water (Fig. 3 A). Remarkably, a lack of editing in FLNA^Q^ mice was associated with severe body weight loss, whereas constitutively FLNA^R^ animals were highly resistant to DSS (Fig. 3 B). The same pattern was seen for colon shortening, a readout for intestinal inflammation, with pronounced shortening in FLNA^Q^ mice, whereas FLNA^R^ mice preserved their colon length (Fig. 3 C). Accordingly, FLNA^Q^ mice presented with a higher histopathological score than FLNA^R^ mice (Fig. 3, D and E), and this was most pronounced in the distal part of the colon, where colitis typically manifests (Koutroubakis, 2010) (Fig. 3, F and G). The pattern of FLNA^R^ protection and FLNA^Q^ sensitivity was observed for most scoring parameters, i.e., the degree of inflammatory cell infiltration, crypt damage, epithelial erosion, and thickening (Fig. 3, H–K). Bulk RNA sequencing of colon tissues from FLNA^R^ and FLNA^Q^ animals on day 10 of DSS-induced colitis (the peak of disease) confirmed that FLNA^R^ mice had less inflammation as indicated by a reduced expression of inflammatory genes (*Il1b*, *Cxcl3*, *Cxcl2*, or *Trem1*) (Weber et al., 2014). FLNA^R^ mice further upregulated genes associated with cell differentiation, survival (e.g., Lgr5, Cbs, and Ascl2), and barrier function (*Cldn2*) (Fig. 3, L and M; and Table S4). Together, a fixed, FLNA^R^ state renders mice highly resistant to DSS-induced colitis, while FLNA^Q^ makes them more susceptible.

### FLNA^R^ reduces early inflammatory cytokine production and intestinal neutrophil accumulation

**Figure 4.**
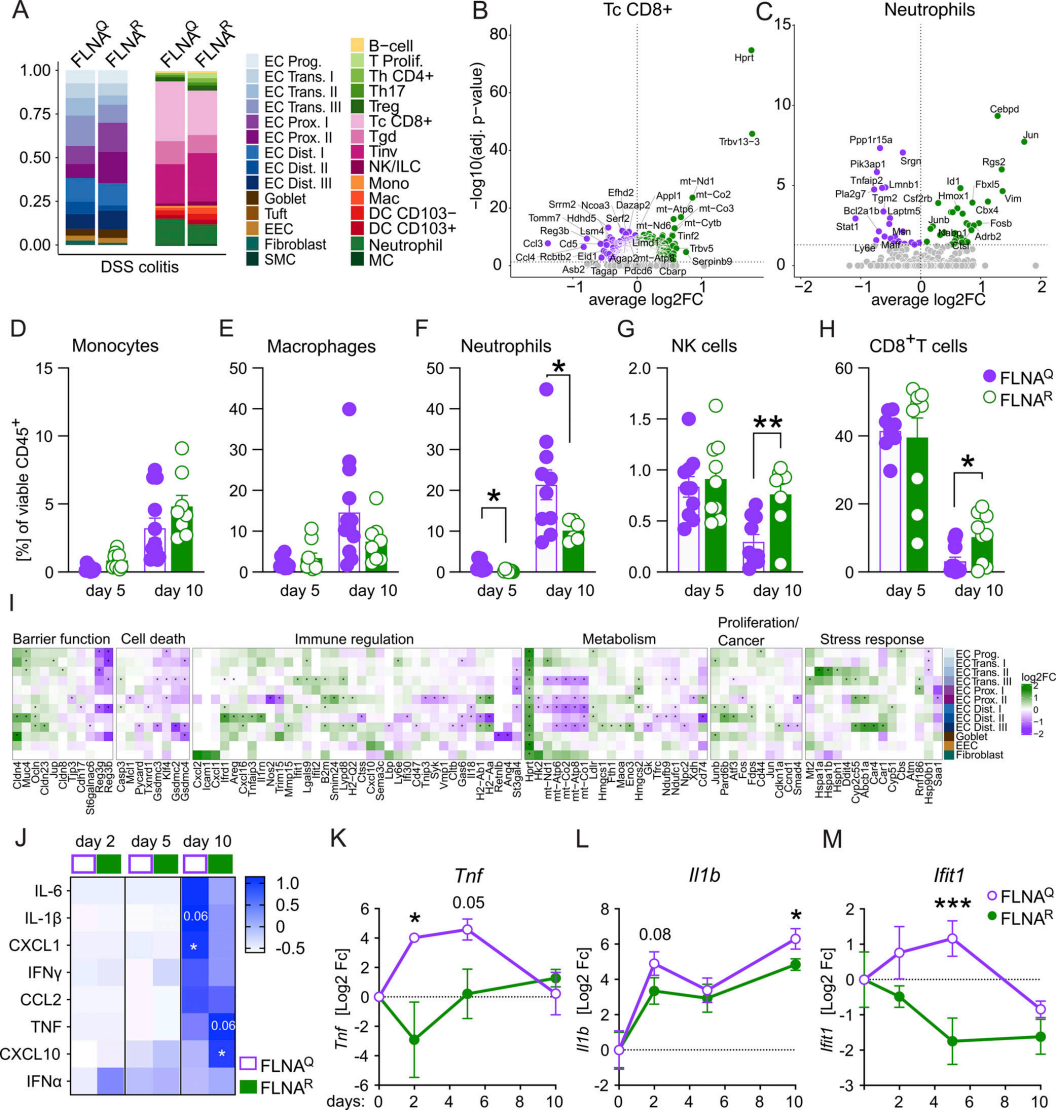
**FLNA** ^**R**^ **reduces early inflammatory cytokine production and intestinal neutrophil accumulation. (A)** Relative numbers of parenchymal (CD45^−^) and immune (CD45^+^) cells in FLNA^R^ and FLNA^Q^ intestines; left panel: EC progenitors (EC Prog.), ECs trans (I–III), proximal ECs (EC Prox. I–II), distal ECs (EC Dist. I–III), goblet cells, tuft cells, enteroendocrine cells (EEC), fibroblasts, and SMCs; right panel: B cells, proliferating T cells (T Prolif.), T helper CD4^+^ (Th CD4^+^), regulatory T cells (Treg), T helper 17 cells (Th17), classical cytotoxic CD8^+^ T cells (Tc CD8^+^), γδT cells (Tgd), invariant T cells (Tinv), NK/innate lymphoid cells (ILC), monocytes (Mono), macrophages (Mac), CD103^−^ and CD103^+^ DCs, neutrophils, and mast cells (MC). **(B and C)** Volcano plots (DEGs, P-adj. ≤ 0.05) of FLNA^R^ versus FLNA^Q^ cells identified by DESeq2 analysis for classical CD8^+^ T cells and neutrophils. **(D–H)** Relative numbers of intestinal monocytes, macrophages, neutrophils, NK cells, and CD8^+^ T cells (Student’s *t* test). **(I)** Heatmaps of selected DEGs in intestinal ECs of FLNA^R^ and FLNA^Q^ mice after 5 days of DSS grouped by cellular processes. Heatmap color represents log_2_-fold increase of expression in FLNA^R^ (green) and FLNA^Q^ (purple). Rows represent cell types of ECs. Asterisks represent significant P-adj. < 0.05 values of log_2_fold change. **(J)** Heatmap of cytokine levels (pg/ml) in mouse colon homogenates shown as z-score across both groups and time points (two-way ANOVA and Šídák multiple comparisons test). **(K–M)** Gene expression analysis by real-time qPCR of FLNA^R^ and FLNA^Q^ colon homogenates. CT values relative to *Gapdh* (2^−ddCT) are shown as log_2_fold change compared with day zero per time point (two-way ANOVA and Tukey’s multiple comparisons test). ***P < 0.001; **P < 0.01; *P < 0.05. Error bars show SEM. Data in A–C and I are from a single experiment (five mice pooled per genotype). Data in D–H are representative from two independent experiments and J–M are from one experiment (*n* = 4–7/genotype and time point).

We next wanted to gain mechanistic insights into the differential DSS susceptibility of FLNA^Q^ and FLNA^R^ mice. Analogous to what we did before in healthy animals, we analyzed single-cell RNA sequencing (scRNA-seq) of viable CD45^+^ and CD45^−^ cells on day 5 of DSS-induced colitis, when mice are not severely sick yet, but colon shortening already manifests in FLNA^Q^ animals (Fig. S2, D and E). An enrichment of fibroblasts and granulocytes and a reduction in goblet and B cells was apparent in both groups upon DSS challenge (Fig. 1 C and Fig. 4 A). In FLNA^R^ animals, we observed a higher frequency of mature EC, which may indicate reduced cell loss and a lower neutrophil abundance (Fig. 4 A). DEGs were determined for all populations except SMC, tuft cells, and proliferating T cells (Table S5).

In contrast to naïve mice, FLNA^R^ and FLNA^Q^ immune cells showed substantial differences during colitis, with most DEGs found in classical cytotoxic CD8^+^ T cells (Tc CD8^+^) (Table S6 and Fig. 4 B). While FLNA^R^ Tc CD8^+^ cells expressed enhanced levels of TCR components (*Trbv13-3* and *Trbv5*), FLNA^Q^ Tc CD8^+^ cells upregulated factors associated with TCR regulation (e.g., *Cd5*) (Azzam et al., 2001), recruitment (*Ccl3* and *Ccl4*), and activation (*Reg3b*) (Honey, 2006; Shindo et al., 2020) (Fig. 4 B). While we also found a substantial number of DEGs in the other two CD8^+^ T cell subsets, other immune cell types were largely unaffected by the FLNA editing status, with one exception: FLNA^R^ neutrophils exhibited a transcriptional profile associated with anti-inflammatory properties (e.g., *Adrb2*, *Hmox1*, and *Rgs2)* (Chan et al., 2018), cell survival (e.g., *Jun*, *Junb*, and *Fos*), and differentiation (e.g., *Id1* and *Csf2rb*) (Buitenhuis et al., 2005). FLNA^Q^ neutrophils, on the contrary, upregulated inflammatory genes (e.g., *Stat1*, *Ly6e*, and *Tnfaip2*) (Jia et al., 2018) (Table S6 and Fig. 4 C). To confirm potential compositional differences and assess immune cell infiltration dynamics, we analyzed cell recruitment over time during colitis. While on day 2 we did not detect increased leukocyte numbers compared with healthy mice, infiltration started to rise by day 5—when also a trend toward increased total leukocyte numbers was found in FLNA^Q^ animals—and got even higher by day 10 (Figure S2F). Cell composition on day 2 was similar to healthy mice, except for a small yet significant increase in monocytes of FLNA^R^ colons (Fig. S1, A and B; and Fig. S2 G). This difference disappeared by day 5 of DSS treatment (Fig. 4 D) and did not translate into altered numbers of macrophages (Fig. 4 E). In accordance with the ScSeq data, neutrophil abundance was lower in protected FLNA^R^ than FLNA^Q^ colons, and this trend continued until day 10 (Fig. 4 F). While FLNA^R^ mice significantly preserved their intestinal lymphocyte pools, a substantial depletion of natural killer cell (NK) and CD8^+^ T cells was observed in FLNA^Q^ mice (Fig. 4, G and H). Other immune cell abundances, such as B cells, Th cells, and DCs, were unaffected (Fig. S2, H–J).

In homeostasis, FLNA^R^ structural cells expressed more genes associated with barrier function (*Reg3b/g*, *Cldn2/8/15*, and *Muc4*) (Fig. 1). During colitis, a higher expression of *Muc4* was maintained, and in addition, *Cldn4*, *Ocl*n, and *Jup*, important tight and adherens junction components, were upregulated (Zhao et al., 2018). *Reg3g*/*3b* were now highly expressed in FLNA^Q^ enterocytes, which may reflect a stronger stimulation of the epithelium by the microbiota (Fig. 4 I) (Shindo et al., 2020). While gasdermin transcription remained higher in FLNA^Q^ cells, some FLNA^R^ ECs increased caspase 3 transcription, suggesting that FLNA^R^ rather promoted a transcriptional state associated with apoptosis, while FLNA^Q^ promoted pyroptotic cell death (Fang et al., 2022). Again, the FLNA editing state affected the expression of proliferation-, cancer-, and stress response–associated genes (*Junb*, *Jun*, *Pard6b*, *Atf3*, *Hspa1a* and -*b*, *Cyp2c55*, and *Abcb1a)* (Nolan et al., 2008; Ku and Cheng, 2020) (Fig. 4 I). We then measured inflammatory cytokines in colon homogenates. Protein cytokine levels were still low on days 2 and 5 but increased by day 10 in both genotypes. Concentrations of IL-1β and the neutrophil chemoattractant CXCL1 were elevated in susceptible FLNA^Q^ mice, while protected FLNA^R^ colons contained more TNF and the IFN-inducible factor CXCL10 (Fig. 4 J). On the RNA level, distinct inflammatory patterns were detectable already as early as day 2, with reduced expression of *Tnf*, *Il1b*, and *Ifit1* in FLNA^R^ compared with FLNA^Q^ mice (Fig. 4, K–M).

Together, we conclude that the FLNA editing state modulates early inflammatory responses to DSS, thus influencing the intestinal cytokine milieu and immune cell infiltration. Considering the importance of neutrophils in driving DSS-induced tissue damage (Li et al., 2020a) and the finding of transcriptional and infiltration differences of neutrophils, we speculated that differential recruitment and function of neutrophils contributed to protection in fully FLNA^R^ animals.

### A potentially protective microbiome does not explain DSS resistance of FLNA^R^ mice

**Figure 5.**
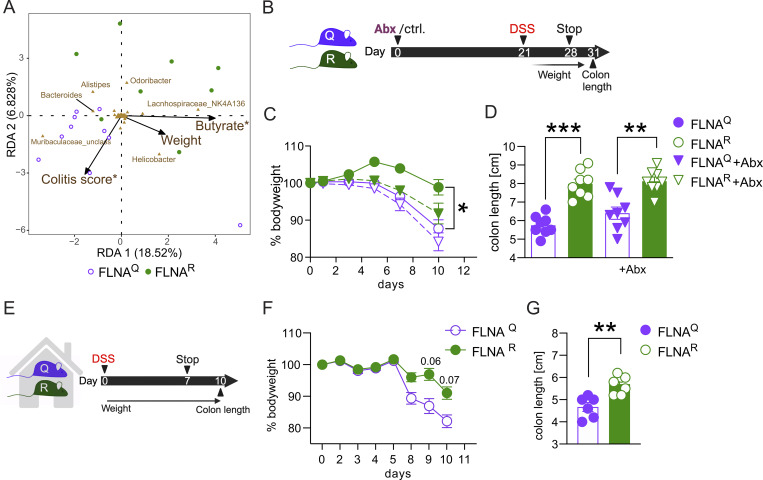
**A potentially protective microbiome does not explain DSS resistance of FLNA** ^**R**^ **mice. (A)** Outcome predictive redundancy analysis (RDA) of the prokaryotic community of FLNA^Q^ (purple dots) and FLNA^R^ (green dots) mice. The relation of the community composition (at the genus level) with the environmental variables: butyrate, colitis score, and body weight loss (% of initial body weight) is shown. Direction and length of arrows show the correlational strength between the abundance of each prokaryotic genus and the environmental variable. Asterisks denote environmental factors that are statistically significant (PERMANOVA; P < 0.05). **(B)** Scheme of antibiotic (Abx) depletion and DSS challenge (*n* = 8 per group). **(C)** Weight loss plotted in % of body weight compared with treatment start for FLNA^R^ and FLNA^Q^ mice with (dashed line) or without (solid line) microbiome depletion. **(D)** Length measurements of colons at day 10 (one-way ANOVA, *F* = 21.20, *DF* = 28). **(E)** Scheme of co-housing and DSS treatment (*n* = 8 per group). **(F)** Weight loss curves for co-housed FLNA^R^ and FLNA^Q^ mice (two-way ANOVA, *F* = 2.941, *DF* = 5). **(G)** Length measurements of colons for co-housed FLNA^R^ and FLNA^Q^ mice at day 10 (Student’s *t* test). ***P < 0.001; **P < 0.01; *P < 0.05. Error bars show SEM. Data in A–G are from one experiment.

**Figure S3.**
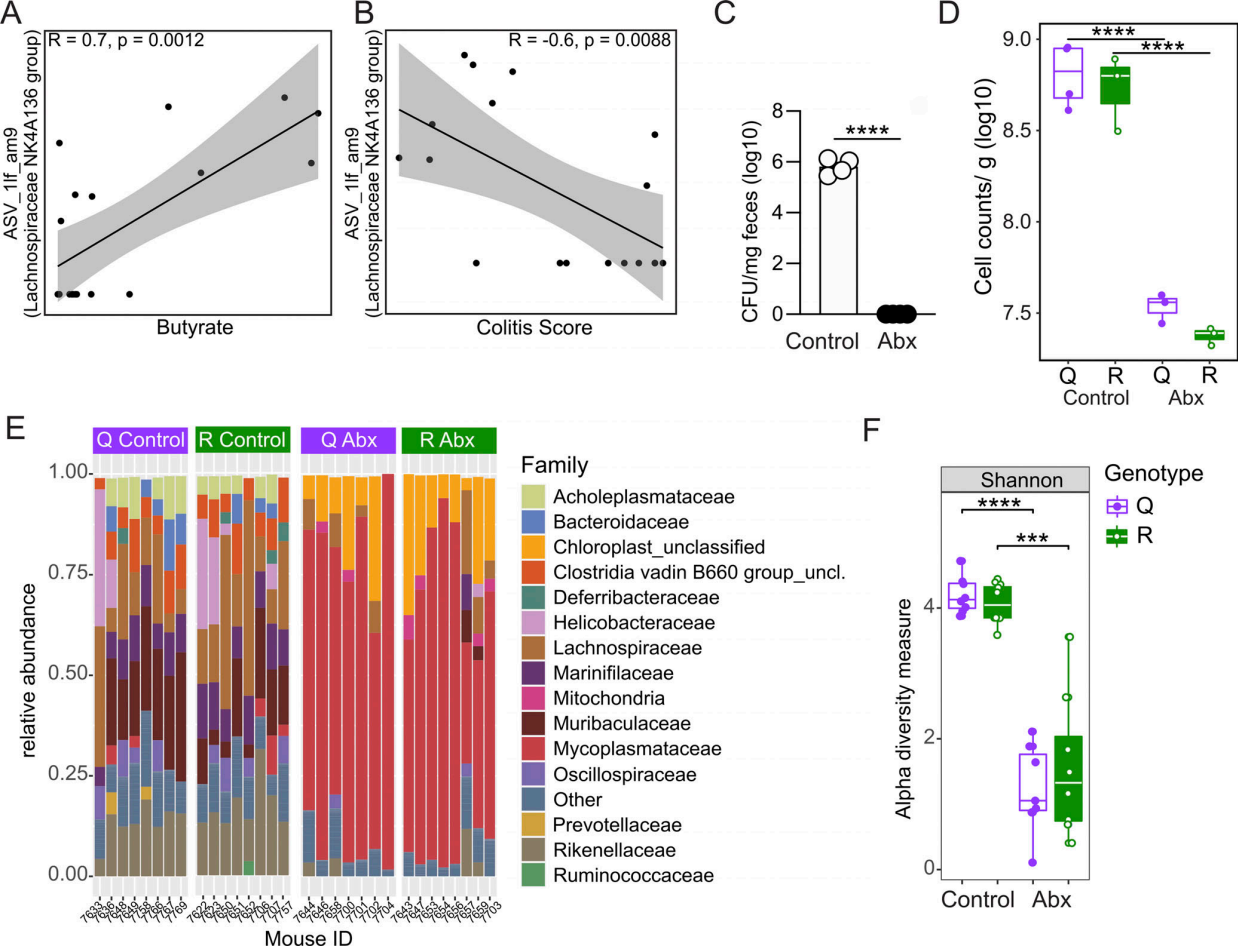
**A potentially protective microbiome does not explain DSS resistance of FLNA** ^**R**^ **mice. (A and B)** Pearson correlation analyses of ASV_1lf_am9 (belonging to the genus *Lachnospiraceae* NK4A136 group) and butyrate concentrations (A) or colitis score (B). **(C)** CFU determination after plating serial dilutions of fecal pellet slurries (*n =* 4 for control and antibiotic [Abx]-treated mice). **(D)** Fecal microbial loads of control (*n* = 4 for FLNA^Q^, *n* = 3 for FLNA^R^) and antibiotic-treated (Abx) mice (open circles*, n* = 3) as assessed by flow cytometry (Welch two sample *t* test). **(E)** Family level relative abundance profiles of gut microbiomes from FLNA^R^ and FLNA^Q^ control or Abx-treated mice. Families with relative abundances <2% are collapsed into the category Other. Each bar represents one mouse. **(F)** Alpha diversity metric (Shannon Index) of samples from FLNA^Q^ and FLNA^R^ control and Abx-treated mice, as assessed by 16S rRNA gene amplicon sequencing analyses (*n* = 7 for Q Abx; *n* = 8 for Q control, R control, and R Abx; Welch two sample *t* test). In D and E, each point represents one mouse, and boxes represent median, first, and third quartiles. Whiskers extend to the highest and lowest values that are within one and a half times the interquartile range. ****P < 0.0001; ***P < 0.001. Data are derived from one experiment.

SCFAs and butyrate in particular have been associated with anti-inflammatory effects in IBD and mouse models of intestinal inflammation (Ranjbar et al., 2021). We thus speculated that the butyrate-enriched microbiome of FLNA^R^ animals may contribute to their DSS resistance (Fig. 2). When we tested if microbiota and butyrate content were associated with the phenotypic outcome of the DSS treatment in FLNA mutant mice, it was not surprising to find a relationship between community composition and fecal butyrate levels (Fig. 5 A). However, the community composition in naïve mice strikingly also predicted the colitis score in FLNA^R^ and FLNA^Q^ mice upon subsequent DSS treatment (Fig. 5 A). Among all genera, the *Lachnospiraceae *NK4A136 group was strongly associated with butyrate levels (Fig. 5 A), likely because organisms from this genus are closely related to several butyrate-producing microbes (Ma et al., 2020). We further identified an amplicon sequencing variant (ASV) within this genus (ASV_1lf_am9, 96% identical to *Lachnoclostridium pacaense*) whose abundance positively correlated with butyrate levels (Fig. S3 A) and negatively with the colitis score (Fig. S3 B). As this suggested a connection between microbiome and colitis severity, we next experimentally tested a potential causality of the microbiome in the DSS resistance of FLNA^R^ mice. First, we depleted the microbiota of the mice for 3 wk with a cocktail of antibiotics (Fig. 5 B), reflected by a reduction in the total number of bacteria (Fig. S3, C and D) and a drop in alpha diversity (Fig. S3 E). However, when microbiome-depleted and control mice were challenged with DSS (Fig. 5 B), FLNA^R^ animals were still significantly and similarly protected from body weight loss and colon shortening (Fig. 5, C and D). In a second approach, we co-housed FLNA^Q^ and FLNA^R^ mice to align their microbiomes prior to the DSS challenge (Fig. 5 E). Co-housing did not abrogate the difference in colitis severity between FLNA mutants, as FLNA^R^ mice still lost less body weight and preserved their colon lengths (Fig. 5, F and G). Thus, the potentially protective microbiome in FLNA^R^ animals can only contribute to, but is not causal for, their protection from DSS-induced colitis.

### FLNA^R^ in myeloid cells protects from colitis

Having excluded a major contribution of the microbiome to the disease phenotype, we next sought to dissect which cell types mediate the differential DSS susceptibility upon fixation of the FLNA editing state. Higher FLNA editing ratios (65–100%) have earlier been shown in mucosal fibroblasts and endothelium and low-medium levels (16–50%) in SMCs and keratinocytes, while immune cells and ECs mainly express FLNA^Q^ under homeostatic conditions (Gabay et al., 2022).

**Figure 6.**
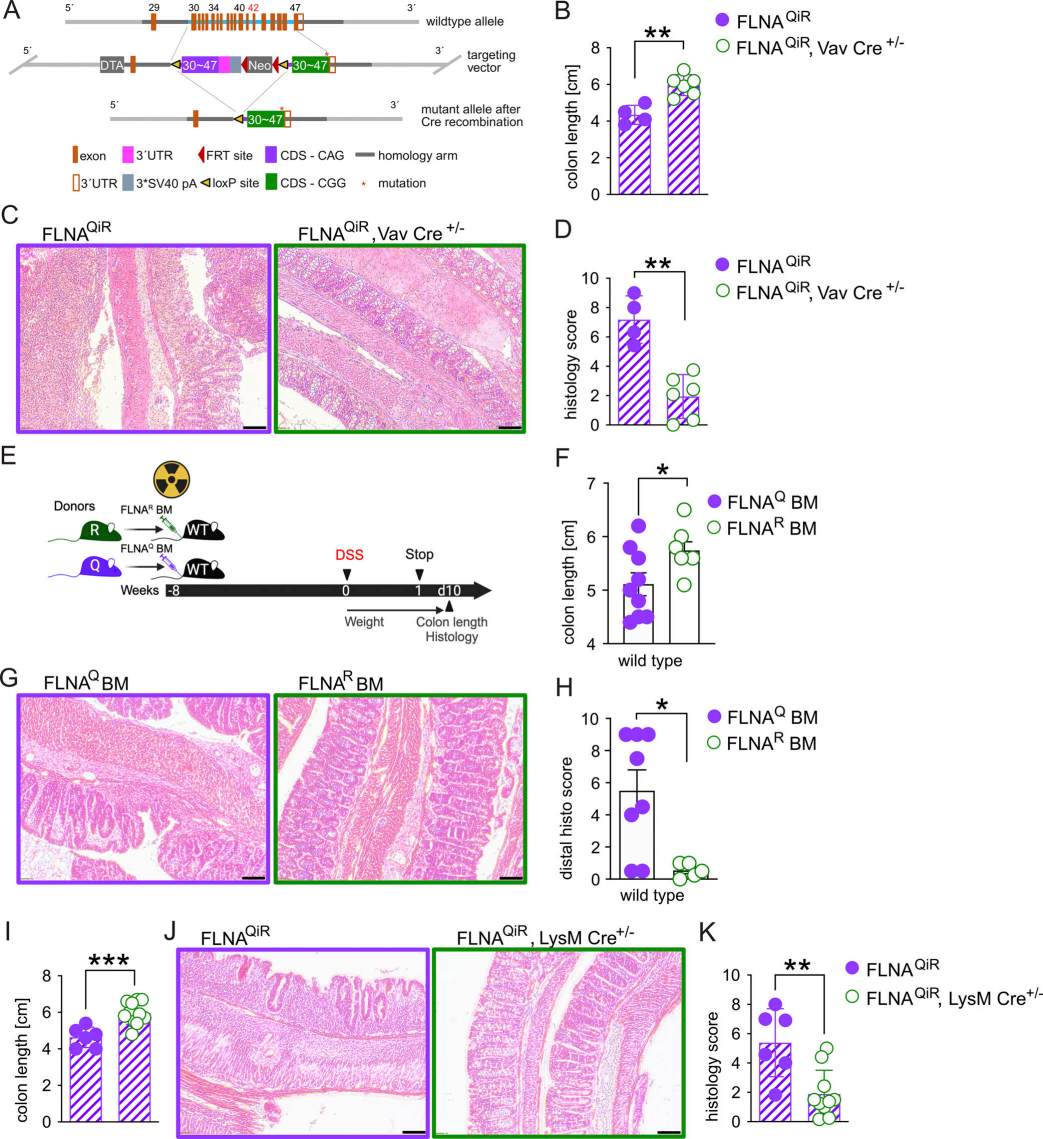
**FLNA** ^**R**^ **in myeloid cells protects from colitis. (A)** Organization of the FLNA WT allele, the targeting vector, and the inducible FLNA^QiR^ allele. The targeting vector cassette replaced exons 30–47 by homologous recombination. The loxP-flanked unedited CDS (purple) and the Neo-cassette are then deleted by Cre-recombinase. **(B–D)** Colon length measurements, representative images of histologic colon sections, and total histology colitis scores of FLNA^QiR^ and FLNA^QiR^ Vav Cre ^±^ mice at day 10 of DSS challenge (*n =* 4 for QiR and *n =* 6 for QiR-Cre, Student’s *t* test and Mann–Whitney *U* test). **(E)** Scheme of BM transplant experiments followed by DSS treatment. **(F–H)** Colon length measurements, representative images of histologic colon sections, and colitis histology score of lethally irradiated WT mice reconstituted with either FLNA^Q^ or FLNA^R^ BM (*n =* 9 for Q and *n =* 6 for R, Student’s *t* test and Mann–Whitney *U* test). **(I–K)** Colon length measurements, representative images of histologic colon sections, and total histology colitis score of FLNA^QiR^ and FLNA^QiR^ LysM Cre ^±^ mice at day 10 of DSS challenge (*n =* 6 for QiR and *n =* 10 for QiR-Cre, Student’s *t* test and Mann–Whitney *U* test). ***P < 0.001; **P < 0.01; *P < 0.05. Error bars show SEM. Scale bars = 50 µm. Data in A–D and I–K are representative of two independent experiments; data in E–H are from one experiment.

**Figure S4.**
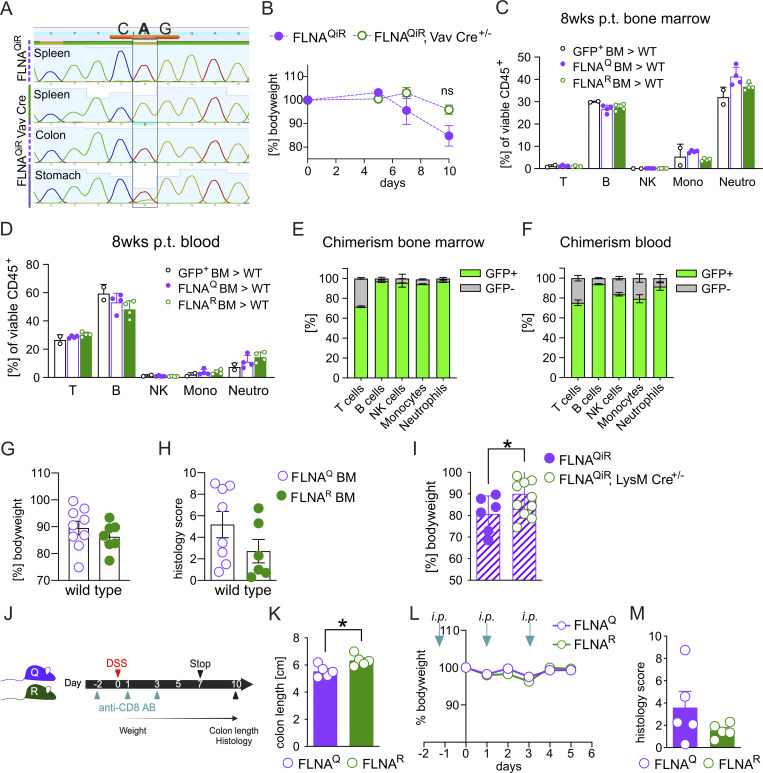
**FLNA** ^**R**^ **in myeloid cells protects from colitis. (A)** Sequencing chromatograms showing the validation of FLNA mutation status on RNA level (“mutant codon” = CAG, with either A or G in the center): RT-PCR products sequenced around the *Flna* editing site (cttcAggga). FLNA^QiR^ mouse tissue (spleen) only expresses FLNA^Q^ (only “A” peak). A change from A to “G” (representing inosine [“I”] in the RNA) occurs only in Cre-recombinase–positive hematopoietic cells (FLNA^QiR^ Vav-Cre in spleen as a tissue enriched in hematopoietic cells compared with colon and stomach tissues). **(B)** Body weight loss curves of FLNA^QiR^ or FLNA^QiR^ Vav-Cre mice after DSS treatment until day 10 (two-way ANOVA). **(C and D)** Leukocyte counts 8 wk after transplantation of control (GFP^+^), FLNA^Q^, and FLNA^R^ BM into WT mice in BM and blood (two-way ANOVA). p.t.=post transplantation. **(E and F)** Chimerism in cell populations 8 weeks after transplantation of control GFP^+^ BM into GFP-WT mice in BM and blood (two-way ANOVA). **(G and H)** Body weight loss and total histopathological colitis score of WT mice transplanted with either FLNA^Q^ or FLNA^R^ BM and subsequently treated with DSS. Weights and scores shown at day 10 (Student’s *t* test and Mann–Whitney *U* test). **(I)** Body weight loss of FLNA^QiR^ or FLNA^QiR^ LysM-Cre mice after DSS treatment at day 10 (Student’s *t* test). **(J)** Scheme for CD8^+^ T cell depletion and DSS challenge (*n* = 5 per genotype). **(K–M)** (K) Colon length measurement of CD8^+^ T cell–depleted FLNA^Q^ and FLNA^R^ mice at day 10 (Student’s *t* test); (L) Body weight loss curves until day 10 (two-way ANOVA); and (M) total histopathological colitis score at day 10 of DSS challenge (Mann–Whitney *U* test). Arrows in L indicate time points of antibody injections. *P < 0.05. Error bars show SEM. Data in A, D–E, and J–M are from one experiment. All other data are representative of two independent experiments.

Considering the high levels of endothelial editing and the significant reduction in intestinal neutrophil accumulation of DSS-resistant FLNA^R^ mice, we next hypothesized that FLNA editing may impact inflammation-induced leukocyte trafficking via its influence on endothelial and/or hematopoietic cells. To investigate this, we used a newly generated mouse, which constitutively expresses FLNA^Q^ but induces FLNA^R^ upon Cre recombination (FLNA^QiR^) (Fig. 6 A). We first generated FLNA^QiR^ Vav1Cre^+/−^ mice to specifically express FLNA^R^ in hematopoietic and endothelial cells (Georgiades et al., 2002) and verified FLNA^R^ expression in the spleen and FLNA^Q^ in tissues with small immune cell populations (intestine and stomach) (Fig. S4 A). When FLNA^QiR^ VavCre^+/−^ and FLNA^QiR^ control animals were subjected to the DSS regime, we found that FLNA^QiR^ VavCre^+/−^ mice were protected, indicated by significantly preserved colon length and an improved colitis score compared with unedited FLNA^QiR^ mice (Fig. 6, B–D). A similar trend was visible for the body weight loss, but this did not reach statistical significance (Fig. S4 B).

To narrow down the effects of a fixed FLNA editing state to endothelial or hematopoietic cells, we next used a bone marrow (BM) transplant approach. To test for major defects of FLNA mutant BM in cell homing and BM reconstitution, we first compared hematopoietic cell repopulation in a setup where we transplanted Ub-GFP (resembling WT), FLNA^Q^, or FLNA^R^ BM into lethally irradiated WT animals. Blood and BM leukocyte counts were similar between groups, and we found no signs of inflammation (Fig. S4, C and D), showing that all genotypes had repopulated similarly by 8 wk after transplantation. As expected, T cells were replaced with the lowest efficiency (around 75–80%), due to the radioresistant properties of certain T cell subsets (Paganetti, 2023). Most efficient replacement was observed for neutrophils and B cells (Fig. S4, E and F). While we cannot directly infer from GFP-Ub chimeras on reconstitution efficiencies in FLNA mutant BM chimeras, we concluded that a fixed FLNA editing state in hematopoietic cells still allows for vital BM reconstitution after irradiation and next transplanted BM from FLNA^Q^ and FLNA^R^ mice into WT mice, followed by DSS challenge (Fig. 6 E). Although we did not observe differences in bodyweight loss (Fig. S4 G), transplantation of FLNA^R^ BM still induced protection indicated by a preserved colon length (Fig. 6 F) and improved colitis scores, particularly in the distal colon (Fig. 6, G and H; and Fig. S4 H). Thus, hematopoietic FLNA^R^ is sufficient to protect from colitis.

Based on this result and the notion that neutrophils may drive potential protective effects—FLNA^R^ neutrophils exhibited an anti-inflammatory transcriptional profile and reduced migration to the tissue during colitis (Fig. 4, C and F)—we crossed FLNA^QiR^ and LysMCre animals. Doing so, we generated mice that either lack editing or express constitutively FLNA^R^ only in myeloid cells, including neutrophils and macrophages (Clausen et al., 1999). Strikingly, FLNA^QiR^ LysMCre^+/−^ mice recapitulated the phenotype we had observed in FLNA^QiR^ VavCre^+/−^ animals and irradiated mice reconstituted with FLNA^R^ BM. FLNA^QiR^ LysMCre^+/−^ significantly preserved colon length (Fig. 6 I) and tissue integrity (Fig. 6, J and K), and additionally, lost less body weight (Fig. S4 I). Notably, CD8^+^ T cell depletion using an established depletion regime with an anti-CD8 antibody (Penttilä et al., 1999) (Fig. S4 J) did not abrogate colon length differences between FLNA^Q^ and FLNA^R^ animals (Fig. S4, K–M). We thus concluded that fixed FLNA^R^ in myeloid cells reduces inflammatory cytokines and neutrophil tissue infiltration during DSS challenge, which drives protection from severe colitis.

### A fixed, fully FLNA^R^ in myeloid cells shifts cellular properties and could be therapeutically exploited in colitis

Having established that a fixed FLNA^R^ state in myeloid cells is protective during DSS-induced colitis, we wanted to follow the dynamics of FLNA editing during colitis in inflamed WT colons.

**Figure 7.**
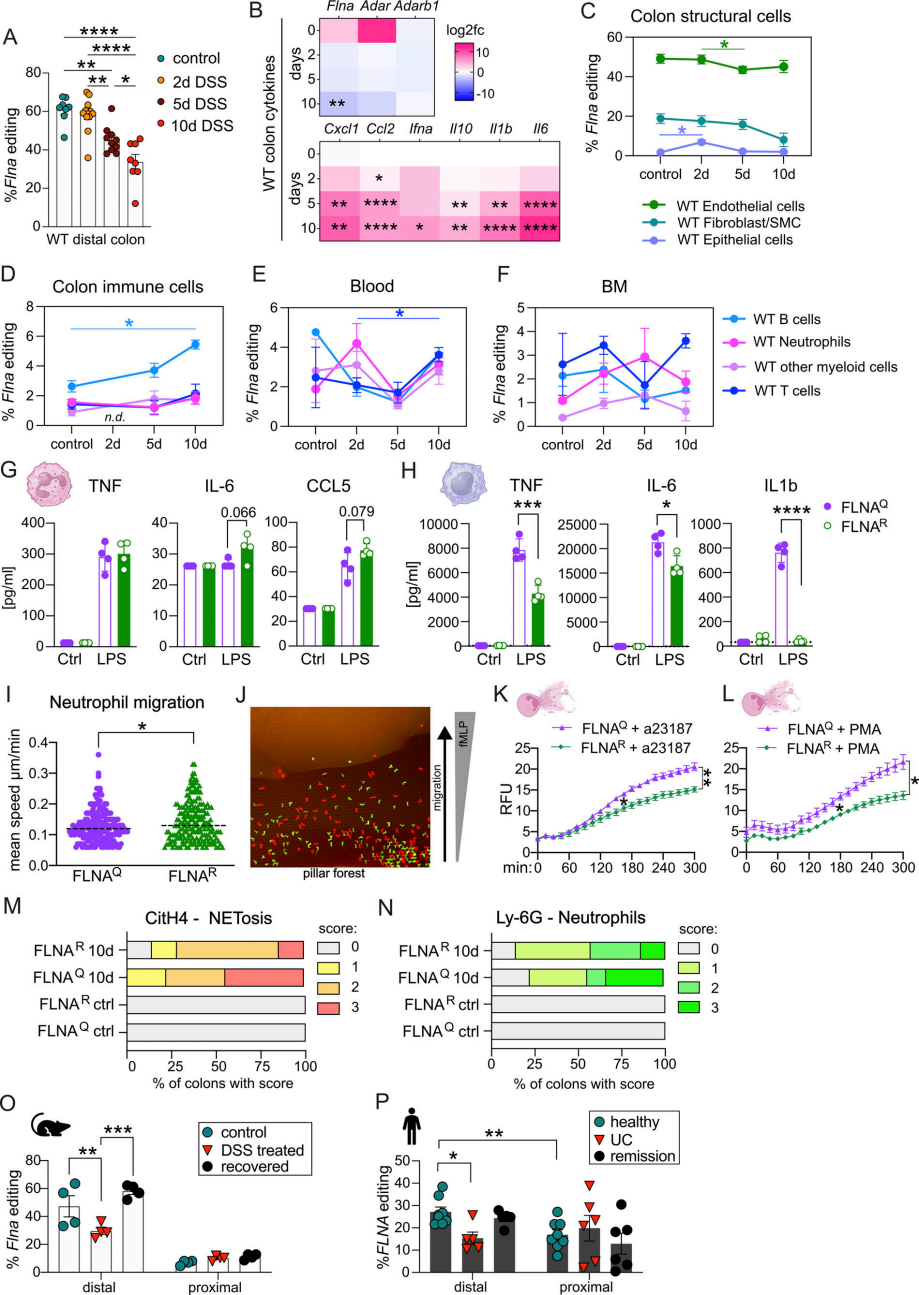
**A fixed, fully FLNA** ^**R**^ **in myeloid cells shifts cellular properties and could be therapeutically exploited in colitis. (A)** *Flna* mRNA editing status of WT whole distal colon was determined by RT-PCR amplicon Illumina sequencing on days (d) 0, 2, 5, and 10 of DSS challenge (one-way ANOVA, *F =* 19.09, *DF* = 3). **(B)** Gene expression analysis by real-time qPCR from WT colon homogenates. CT values relative to *Gapdh* (2^−ddCT) are shown as log_2_fold change compared with day zero depicted as heatmap at different time points of DSS treatment (mixed-effects analysis followed by Tukey’s multiple comparisons test). **(C–F)***Flna* mRNA editing status of sorted cell populations of two–six pooled WT mouse colons, BM, and blood determined by RT-PCR amplicon Illumina sequencing on days 0, 2, 5, and 10 of DSS challenge (mixed-effects analysis followed by Tukey’s multiple comparisons test). **(G and H)** Bar graphs of cytokine levels (pg/ml) in supernatants from LPS-stimulated mouse neutrophils and BMDMs (Student’s *t* test). **(I)** Neutrophil migration speed in a pillar forest of a microfabricated PDMS device toward an fMLP gradient (Student’s *t* test). Dashed lines represent the median. **(J)** Exemplary image of CSFE- and TAMRA-stained FLNA^Q^ and FLNA^R^ neutrophils within the device. **(K and L)** Quantification of DNA release as relative fluorescence units to measure NETosis of isolated neutrophils after stimulation with a23187 or PMA over time. Time course data were plotted in a line graph with mean ± SEM (two-way ANOVA followed by Tukey’s multiple comparisons test, *F* = 11.94, *DF* = 60). **(M and N)** Scoring of histological colon sections stained for NETs by anti-citrullinated histone 4 (citH4) or neutrophils by anti-mouse Ly-6G. **(O)***Flna* editing levels in intestinal tissues of WT mice: healthy mice, mice with acute colitis (day 10 of DSS), or mice recovered from colitis (day 21) were analyzed for *Flna* editing levels in distal and proximal colon separately (*n =* 4 per time point; one-way ANOVA, *F* = 9.208, *DF* = 18). **(P)***FLNA* editing levels in human colon biopsies of healthy donors (*n =* 9), patients suffering from active UC (*n =* 6), and patients in remission from active UC (*n =* 6). Distal and proximal colon biopsies were analyzed separately (one-way ANOVA, distal: *F* = 7.326, *DF* = 15); comparison healthy-distal versus healthy-proximal (Student’s *t* test). ****P < 0.0001; ***P < 0.001; **P < 0.01; *P < 0.05. Error bars show SEM. Data in A–F are pooled from two independent experiments (*n* = 3–5 pooled colons per group, per experiment). Data in G–L are representative of two experiments, data in M and N are pooled from two experiments, and data in O are from a single experiment.

**Figure S5.**
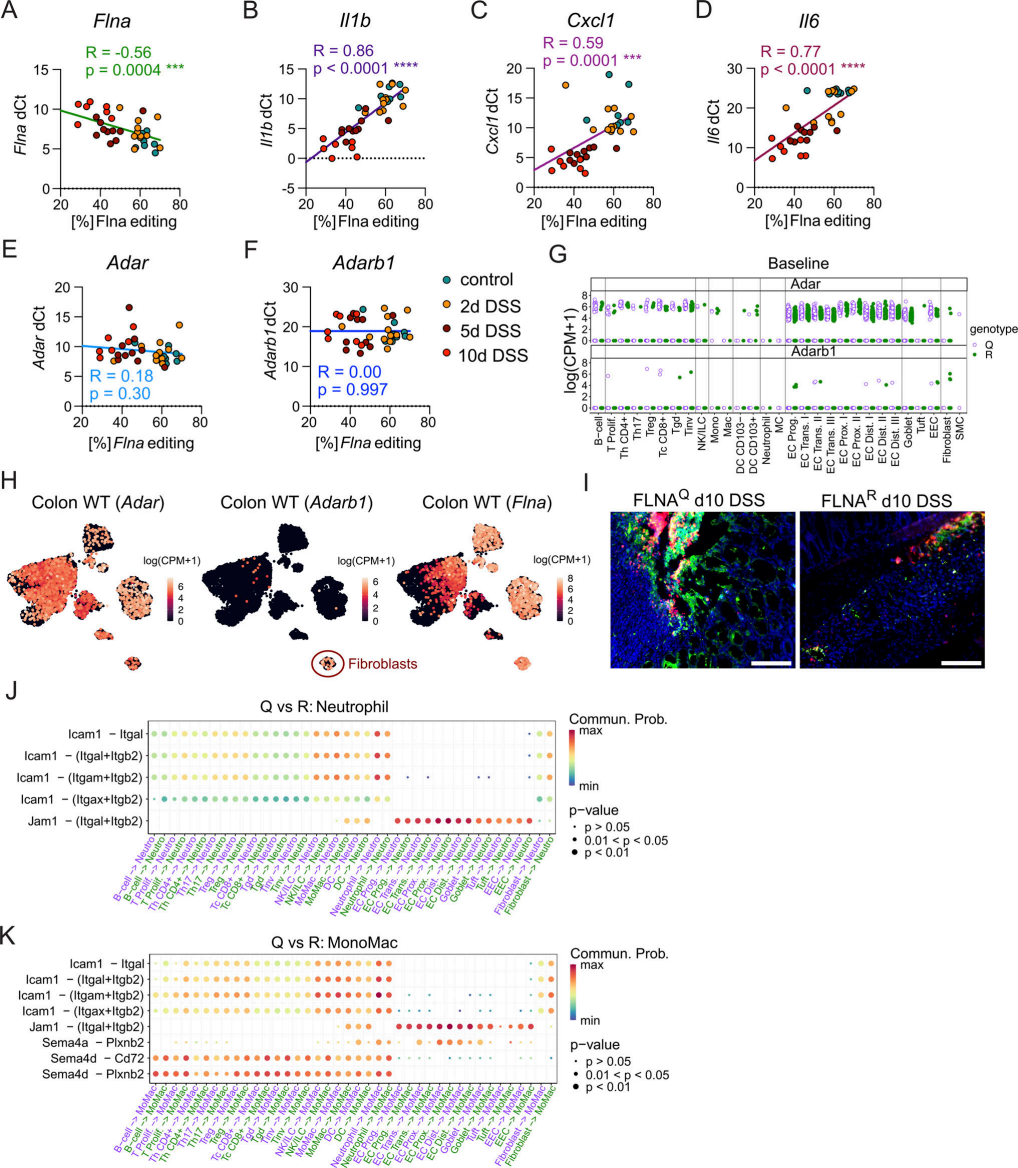
**FLNA** ^**R**^ **in myeloid cells protects from colitis. (A–F)** Correlations of delta C_T_ values from RT-qPCR analysis of mRNA expression versus FLNA editing levels determined by amplicon sequencing analysis in distal colon on days: 0, 2, 5, and 10 (simple regression analysis). Higher dC_T_ = lower expression. **(G)** Dot plots representing *Adar* and *Adarb1* expression in log counts per million [log(CMP+1)] in untreated FLNA^Q^ and FLNA^R^ mouse colons determined by scRNA-seq in different cell populations. **(H)** Expression levels of *Adar* and *Adarb1* in WT colon cells analyzed by scRNA-seq and shown as heat gradients in the clustered cells [log(CMP+1)]. **(I)** Representative images of colon histologic sections stained for NETosis (by CitH4 AB—red) and neutrophils (by Ly-6G AB—green) at day 10 of DSS in FLNA^Q^ (left) and FLNA^R^ (right); DAPI in blue. Scale bar = 100 µm. **(J and K)** Selected interactions quantified by CellChat analysis of scRNA-seq data of FLNA^Q^ and FLNA^R^ mouse colons after 5 days of DSS challenge. Communication probabilities (interaction strength) of different cell types to neutrophils (J) and monocytes/macrophages (K). Data in A–F are from one experiment (*n* = 8–11 per time point). For G and H, data are from a single experiment (cells from 3–5 WT colons were pooled for ScSeq). Data in I are representative of two experiments and data in J and K are from a single experiment.

Using amplicon sequencing, we first assessed FLNA editing in distal colon tissue in homeostasis, on days 2, 5, and 10 of acute colitis. FLNA was edited to 60% at baseline, and this was maintained on day 2 after DSS challenge. From day 5 on, the editing ratio decreased until day 10, where we only found editing of 30% (Fig. 7 A), and this went along with a reduction of *Flna* expression (Fig. 7 B upper panel and Fig. S5 A). Reduced editing and *Flna* expression levels were associated with an increased expression of classical proinflammatory cytokines and chemokines like *Il1b*, *Cxcl1*, or *Il6*, which are typically induced during colitis (Fig. 7 B lower panel, Fig. S5, B–D). Expression levels of *Adarb1—*encoding for ADAR2 that catalyzes *Flna* mRNA A-to-I deamination (Stulić and Jantsch, 2013)—did neither correlate with *Flna* expression nor editing levels and remained low but stably expressed during colitis (Fig. 7 B lower panel and Fig. S5 F). A trend for decreased Adar (ADAR1) expression was observed, but it did not correlate with *Flna* editing levels (Fig. 7 B lower panel, Fig. S5 E). Together, these data suggested that the downregulation of FLNA editing during colitis was not caused directly by a reduction of the editase (ADAR2) but rather associated with inflammation and, most likely, tissue accumulation of unedited immune cells and/or loss of highly edited cells.

To address which cells FLNA^R^, we took the challenge to sort different structural and immune cell populations from colons of healthy and DSS-challenged animals. As seen in our scRNA-seq datasets, our initial colon preparation protocol was optimized for the isolation of intraepithelial lymphocytes, lamina propria immune, and intestinal ECs (Campbell et al., 2020), but failed to obtain viable intestinal endothelial cells. We therefore performed separate experiments from which we isolated CD31^+^ intestinal endothelial cells and a CD45^−^ EpCam^−^ CD31^−^ cell fraction enriched for fibroblasts and SMCs. ECs expressed only low levels of FLNA^R^. Higher levels (up to 50%) were found in endothelial and moderate levels in the fibroblast/SMC fraction (up to 20%). Endothelial cells and fibroblast/SMCs reduced their editing frequencies during colitis, although changes for fibroblast/SMCs did not reach statistical significance (Fig. 7 C). Analysis of intestinal neutrophils on day two after DSS challenge was hampered by insufficient cell numbers retrieved (even upon pooling of multiple colons). We thus alternatively sorted immune cells from blood and BM. Editing was generally low, but not absent, in isolated intestinal immune cell populations, namely T cells, B cells, neutrophils, and a fraction enriched for CD11b^+^ myeloid cells other than neutrophils. Except for a significant elevation of FLNA editing in B cells by day 10 of DSS colitis, editing fluctuated between 0% and 5% and was not affected by treatment (Fig. 7 D). A similar result was found in blood and BM (Fig. 7, E and F). Accordingly, and in contrast to ubiquitously expressed *Adar*, we only found high levels of *Adarb1* in fibroblasts and some few enterocytes, while it was not detectable in most immune cells in FLNA^Q/R^ colons and in the ScSeq dataset derived from WT animals (Fig. S5, G and H). Together, these data showed that a fixed FLNA^R^ state in neutrophils and other myeloid cells conferred protection from colitis, despite a low FLNA editing ratio in these cells, whereas, similar to other tissues (Gabay et al., 2022), FLNA is mainly edited in colon endothelium, fibroblasts, and SMCs. We thus propose that targeted myeloid cell FLNA editing reveals a new option of therapeutic intervention for intestinal inflammatory diseases.

To better characterize the consequences of an artificial FLNA^R^ state in myeloid cells, we assessed the functional impact of FLNA^R^ on critical myeloid cell effector functions using primary BM neutrophils and BM-derived macrophages (BMDMs) isolated from FLNA^Q^ or FLNA^R^ mice. Upon stimulation with the TLR 4 ligand, LPS, neutrophils of both genotypes produced even amounts of TNF, but FLNA^R^ neutrophils showed a trend to release more of the inflammatory cytokine IL-6 and the chemokine CCL5 (Fig. 7 G). In BMDMs, which are much more potent cytokine producers than neutrophils, we interestingly found the opposite effect. FLNA^R^ BMDMs were significantly hampered in TNF, IL-1β, and IL-6 release in response to LPS as compared with FLNA^Q^ BMDMs (Fig. 7 H), showing that even amongst myeloid cells, FLNA editing exerts highly cell type–specific effects. Using microfluidic chambers, we assessed neutrophil migration toward an fMLP gradient. While both editing states allowed for directed migration of neutrophils, FLNA^R^ neutrophils migrated slightly, yet significantly, faster than FLNA^Q^ neutrophils. However, this difference was only measurable during the first hour of observation (Fig. 7, I and J), before extreme collective dynamics (swarming behavior) masked cell-autonomous differences. It remains elusive whether such subtle differences between the genotypes could impact physiology. Neutrophil extracellular trap (NET) release, the controlled expulsion of extracellular fibers containing DNA, histones, and antibacterial granule components, is a key feature of activated neutrophils in inflammatory settings (de Bont et al., 2019). During acute infection, NETosis can hinder pathogen dissemination but promotes immunothrombosis, inflammation, and tissue damage (dos Santos Ramos et al., 2021). In return, inflammatory mediators such as IL-1β and TNF can boost NET release (Tall and Westerterp, 2019; Neuenfeldt et al., 2022). While we had observed a rather enhanced activity of FLNA^R^ neutrophils regarding cytokine release and migration, we strikingly found that FLNA^R^ neutrophils were less prone to release NETs in response to a23187 and PMA, two established NET inducers (Kenny et al., 2017) (Fig. 7, K and L). Accordingly, we found reduced NET formation in vivo during colitis, suggesting that a fixed FLNA^R^ state in myeloid cells may promote a micromilieu that contains inflammation and NETosis (Fig. 7, M and N; and Fig. S5 I).

To foster the mechanistic interpretation of our findings, we investigated potential differences in cell–cell interaction using CellChat analysis based on our single-cell datasets (Jin et al., 2021). This revealed similar signaling patterns (with regard to the inferred signaling pathways) but distinct interaction strengths between FLNA^Q^ and FLNA^R^ cell populations. In particular, the interaction of the intracellular adhesion molecule-1 (ICAM1) with different integrins seemed affected by the FLNA editing state. While the differences are rather subtle, they were most pronounced in myeloid cells, with FLNA^Q^ neutrophils showing stronger autocrine signaling and an increased interaction with DCs and MonoMacs (a mixed population of monocytes and macrophages). In contrast, FLNA^Q^ fibroblast signaling to neutrophils via the ICAM1-integrin axis was reduced (Fig. S5 J). FLNA^Q^ and FLNA^R^ MonoMacs differed in their signaling through semaphorin 4D (Sema4D/CD100), an immunoregulatory transmembrane protein signaling to Cd72 or plexins in immune and nonimmune cells, respectively (Suzuki et al., 2008). The interaction of macrophage Sema4d and Cd72 on other immune cells was more pronounced for FLNA^R^ cells. Similar to neutrophils, ICAM1-integrin signaling was slightly enhanced from FLNA^Q^ immune cells to MonoMacs, while reduced from FLNA^Q^ fibroblasts to MonoMacs (Fig. S5 K). Although these results are purely predictive, they may explain increased NETosis of FLNA^Q^ neutrophils (Fig. 7, K and L). ICAM1^+^ neutrophils have been shown to be more prone to ICAM1-RhoA–mediated NETosis, which relies on Rho kinase-dependent actin remodeling (Ode et al., 2018). Along with this, we have found previously that FLNA^Q^ cells show mislocalization of p190Rho-GAP and increased levels of activated RhoA-GTP (Jain et al., 2018). In macrophages (MonoMac), enhanced Sema4d-Cd72 signaling may promote their phagocytic capacity (Galuppo et al., 2018), which could affect cytokine production and the removal of bacteria.

We lastly asked how FLNA editing recovers after acute colitis and compared control, acute DSS colitis (day 10), and samples from recovered animals (day 21). We found FLNA editing levels to be significantly reduced during acute disease (Fig. 7 O). Notably, humans showed the same dynamic with reduced FLNA editing levels in intestinal biopsies from patients suffering from acute UC as compared with healthy controls or patients in remission (Fig. 7 P). Further, editing levels of mice and humans were most pronounced in the distal colon (Fig. 7, O and P), highlighting the strong conservation of FLNA editing across species.

Together, we found that mice expressing only FLNA^R^ were highly protected from colitis, while a lack of FLNA editing rendered them more sensitive. DSS resistance is transplantable and reproducible in conditional, myeloid cell–specific FLNA^QiR^ LysMcre^+/−^ animals. Targeted introduction of an FLNA^R^ state in myeloid cells reduces intestinal inflammation and colitis severity, likely via its influence on neutrophils and inflammatory properties of macrophages. Our data also show a similar dynamic of FLNA editing during acute colitis and recovery in mice and humans and thus highlight the therapeutic potential of targeted FLNA^R^ induction in IBD.

## Discussion

We show that the FLNA editing state is a key determinant of colitis severity. Physiologically, FLNA is strongly edited in the distal colon, precisely in fibroblasts, smooth muscle cells, and endothelial cells, and this is lost during acute colitis in mice and humans. Accordingly, mutant mice expressing only FLNA^R^ were DSS resistant, whereas FLNA^Q^ animals were hypersensitive. A fixed FLNA editing state widely affected transcriptional states of intestinal structural and immune cells and altered the microbiome composition. Using cohousing, depletion, transplantation, and transgenic mouse approaches, we find that protection via FLNA^R^ is largely caused by its immunomodulatory properties in myeloid cells, likely neutrophils and macrophages. Our study thus reveals novel insights into the modulating properties of FLNA editing states in the colon and highlights potential benefits of targeted, site-directed FLNA editing (SDRE) in myeloid cells as a novel therapeutic approach for IBD.

Mice with a fixed FLNA^Q^ or FLNA^R^ state exhibit profound differences in colon epithelial and CD8^+^ T cell transcriptomes. Together with a potentially protective, immunomodulatory microbiome with more butyrate (Ranjbar et al., 2021) in FLNA^R^ mice, it was tempting to speculate that FLNA^R^ affected colitis susceptibility via the cross talk between epithelial and T cells with the microbiota. This was supported by a differential expression of defensins (*Reg3b* and *Reg3g*) between FLNA^R^ and FLNA^Q^ ECs and CD8^+^ T cells. *Reg3b* and *Reg3g* are protective in different settings of intestinal inflammation (Bajic et al., 2020; Shindo et al., 2020) and typically get induced by TLR activation (Udomsopagit et al., 2020). However, in two different experimental approaches, we could not prove a causal connection between microbiome composition and DSS sensitivity, suggesting that distinct microbiomes are likely a consequence of genotype-specific host intrinsic changes. In spite of FLNAs importance for endothelial permeability (Bandaru et al., 2021) and observed differences in claudin and mucin-4 expression, editing states did not impact epithelial barrier integrity. As the contribution of different claudins and mucin-4 to IBD pathogenesis is still poorly understood (Čužić et al., 2021), we conclude that FLNA editing modulates specific tight junction components but cumulatively does not alter barrier leakiness.

Aside from its essential functions as a cytoskeleton cross-linker, FLNA interacts with key regulators of cellular signaling like Syk or R-Ras (Nakamura et al., 2011). Genetic fixation of an FLNA^Q^ or FLNA^R^ state affected the expression of genes involved in proliferation, differentiation, and survival signaling like AP-1 complex components (*Jun*, *Fos*, and *Atf3*) (Chinenov and Kerppola, 2001) or *Hprt* (Wang et al., 2021) across different cell types, indicating that FLNA editing may adapt cellular responses to growth factors or cytokines. Importantly, these effects were prominent in structural cells but also present in neutrophils during colitis, indicating cell type–independent features of FLNA^R^. Syk promotes STAT1 activation (Liu et al., 2021), and as FLNA^Q^ neutrophils upregulated genes associated with inflammation like *Ly6e*, *Tnfaip2*, and *Stat1* itself (Jia et al., 2018), FLNA editing might suppress STAT1 activation. FLNA-dependent Syk regulation downstream of the TCR (Aksentijevich, 2021) may also explain transcriptional differences in the CD8^+^ T cell compartment. However, CD8^+^ T cell depletion did not abrogate protection in FLNA^R^ animals, and protection was driven by FLNA^R^ in lysozyme expressing, i.e., myeloid cells.

When investigating physiological FLNA editing, we found high levels only in the distal colon and could attribute this largely to endothelial cells and a cell fraction containing fibroblasts and SMCs. Of note, fibroblast/SMC and endothelial cell isolations were done from the whole colon and not only from the distal part, which may account for discrepancies between editing frequencies in distal colon tissue and sorted cells. A limitation of our study is the lack of an endothelial cell population in our single-cell sequencing dataset, caused by differential technical requirements to obtain healthy epithelial, endothelial, and immune cell fractions. However, we can exclude a strong contribution of endothelial FLNA^R^ to the DSS resistance phenotype due to its transplantability and its prevalence in FLNA^QiR^ LysMCre^+/−^ mice, which exclusively express FLNA^R^ in myeloid cells. Another limitation of the study is a lack of direct comparisons of FLNA^R^, FLNA^Q^, and variably edited WT mice. This was not feasible due to the huge impact of breeding and cage effects on intestinal homeostasis and—even more pronounced—DSS susceptibility. In our experience and according to the literature, littermate breeding is a critical requirement in studies using murine IBD models, which are particularly sensitive to cage, microbiota, and genetic effects (Singh et al., 2021; Robertson et al., 2019; Mamantopoulos et al., 2017). While we would have preferred a setup that allows us to draw conclusions about fixed editing states compared with physiologic editing, it is not possible to obtain littermates from all three genotypes. We thus refrained from including WT animals in most of our setups and compared the two fixed states of FLNA, which—we believe—is more accurate.

Since immune cells—in colon, blood, and BM—only exhibit low-grade editing in all tested conditions, our finding that protection from colitis is driven by myeloid FLNA^R^ appeared counterintuitive at first. However, FLNA impacts cell migration and adhesion by regulating surface integrin expression and by linking integrins to the actin cytoskeleton (Lamsoul et al., 2020). While FLNA deficiency is compensated by FLNB in many cell types (Baldassarre et al., 2009), FLNA deficiency rendered specifically neutrophils more adhesive by enhancing their interaction with integrins (Uotila et al., 2017). While we found reduced numbers of infiltrating neutrophils in the intestinal tissues of protected FLNA^R^ animals, we did not find a defect in FLNA^R^ neutrophil migration in vitro. In contrast, we found that FLNA^R^ neutrophils were slightly faster at the onset of chemotaxis assays (1 h). However, in later phases, this subtle defect was masked by the extreme temporal dynamics neutrophils develop at high population densities (so-called swarming behavior) (Palomino-Segura and Hidalgo, 2020). Hence, it seems unlikely that such subtle differences impact pathology.

In neutrophils, FLNA was shown to regulate reactive oxygen production and NET formation (Uotila et al., 2017), key antibacterial effector functions and drivers of tissue damage (Burn et al., 2021). We found protective FLNA^R^ neutrophils produced less and FLNA^Q^ neutrophils more NETs in vitro. Along with this, FLNA^R^ neutrophils showed downregulation of *Lmnb1*, which has been shown to critically influence nuclear envelope rupture during NET formation (Li et al., 2020b; Saunders and Parent, 2020). Counterintuitively, FLNA^R^ neutrophils appeared slightly more reactive to TLR stimuli. However, even though we did not find relevant transcriptional changes in macrophages, FLNA^R^ BMDMs showed an impaired capacity to produce IL-6, IL-1β, and TNF upon TLR stimulation in vitro. Notably, macrophages, more than neutrophils, shape the inflammatory microenvironment of the colon (Chen et al., 2023; Ren et al., 2023), which in turn is strongly influencing neutrophil NETosis (Tall and Westerterp, 2019; Neuenfeldt et al., 2022). Furthermore, macrophage migration in dense environments was shown to rely on FLNA, and targeted reduction of FLNA impairs macrophage functions in an atherosclerosis model (Bandaru et al., 2019; Guiet et al., 2012). We thus propose a model in which, upon targeted editing in myeloid cells, FLNA^R^ in macrophages dampens their inflammatory responses to the microbiota upon DSS treatment, which in turn leads to a reduced recruitment of FLNA^R^ neutrophils. The already reduced inflammatory milieu and the intrinsic feature of FLNA^R^ neutrophils to be less prone to NETosis then mediate tissue protection and resistance to DSS-induced colitis. This protection may be further amplified by a protective microbiome and anti-inflammatory transcriptional state of the epithelium in a background with a complete FLNA^R^ state. Reduced editing is found in humans during acute colitis, which increases upon remission. Thus, due to the high conservation of FLNA editing in terms of local levels and changes thereof during acute disease in mice and humans, SDRE of FLNA in myeloid cells may be exploited as a future therapeutic intervention for immunomodulation in settings of acute intestinal inflammation.

## Materials and methods

### Human material

Human intestinal biopsies were collected after written consent was obtained during regular colonoscopies at the Division of Gastroenterology and Hepatology of the Medical University of Vienna. In total, biopsies of 21 patients were collected: samples of nine healthy donors, six active UC, and six colitis patients in remission, each with a proximal and distal biopsy. Clinical data and treatment regimens are summarized in Table S7. Samples are listed in Table 1. This study was conducted under the Health Research Authority of the Ethics Committee of the Medical University of Vienna, ethics approval number (“Ribonucleic acid variation in the intestine,” Ethics Committee number (EK-Nr): 1692/2020, voted positively on 17 Sept. 2020, granted to C. Gasche).

### Mice

All animal experiments in this study were performed in compliance with the guidelines of the Ethical Review Committee of the Medical University of Vienna and national laws (approval numbers issued by the Austrian authorities: BMWFW-66.009/0260-WF/V/3b/2016 and 2021-0.203.240). All animals were used at an age of 8–12 wk.

FLNA^Q^, FLNA^R^, IL-10 KO, Vav-Cre, and LysMCre mice were previously described (Jain et al., 2018, 2022a; de Boer et al., 2003; Kiesler et al., 2015; Clausen et al., 1999). IL-10 KO mice were interbred with FLNA^R^ and FLNA^Q^ animals to obtain littermates of FLNA^WT^–IL-10^+/+^, FLNA^WT^–IL-10^−/−^, FLNA^Q^–IL-10^−/−^, and FLNA^R^–IL-10^−/−^.

Inducible FLNA^QiR^ mice were generated for this study by Taconic Biosciences (formerly Cyagen). This mutant strain harbors an FLNA^Q^ locus and an adjacent sequence encoding for a pre-edited FLNA^iR^ sequence to be exchanged with the FLNA^Q^ locus (Fig. 6 H). More specifically, exons 30–47 (National Center for Biotechnology Information [NCBI] Reference Sequence: XM_011247549.3; ATG start codon in exon 2, stop codon in exon 47, based on Transcript: ENSMUST00000114299) were replaced with a cassette containing the *Flna* CDS encoding FLNA^Q^, flanked by loxP sites plus the mutant CDS (loxP-endogenous SA of intron 29-CDS of exon 30∼47-3′UTR-3*SV40 pA-FRT-Neo cassette-FRT-loxP-endogenous SA of intron 29-mutant CDS of Exon 30–47). Thus, in these mice, Cre-recombination replaces the FLNA^Q^ (last codon in exon 42 = CAG) with the FLNA^iR^ CDS (last codon in exon 42 = CGG). A targeted ES cell clone was injected into C57BL/6 albino embryos, which were reimplanted into CD-1 pseudopregnant females. Founder FLNA^QiR^ animals were identified by their coat color, and their germline transmission was confirmed by breeding with C57BL/6 females and subsequent genotyping of the offspring. FLNA^QiR^ Vav-Cre^+/−^ and FLNA^QiR^ LysMCre^+/−^ mice, with expression of pre-edited FLNA^iR^ in hematopoietic/endothelial cells or myeloid cells, and FLNA^QiR^ littermate controls were generated by crossing FLNA^QiR^ and Vav1Cre^+/−^ or LysMCre^+/−^ animals, respectively. Of note, FLNA^QiR^ Vav1Cre^+/−^ and FLNA^QiR^ LysMCre^+/−^ mice were born at normal Mendelian ratios, appeared healthy, and did not show any obvious clinical phenotypes. All mice were bred on a C57BL/6 background and maintained in the specific pathogen–free animal facility of the Medical University of Vienna (22°C, 12 h light/12 h dark cycle), with free access to food and sterile water.

### Murine colitis models

DSS-colitis was induced in 8–10-wk-old mice by adding 2–2.5% DSS in the drinking water for 7 days, followed by 3 days with regular drinking water. Body weight and health status of the animals were monitored throughout the experiment. Mice were euthanized on day 5 or 10 by i.p. Ketazol/Rompun overdose. At the endpoint, colons were isolated, measured, and prepared for histology, RNA isolation, FACS analysis, or sorting.

The development of spontaneous colitis in FLNA^WT^–IL-10^+/+^, FLNA^WT^–IL-10^−/−^, and FLNA^Q^–IL-10^−/−^ mice was assessed by monitoring the body weight twice per week after weaning. All animals were euthanized at the time of onset of colitis symptoms at 9–10 wk of age. Body weight and colon length were measured after sacrifice, and the histological sections of colon rolls were scored for colitis parameters.

### Microbiome depletion

For antibiotic treatment, mice were randomly assigned to two groups. One group received a cocktail of antibiotics (1 mg/ml ampicillin, 1 mg/ml vancomycin, 1 mg/ml streptomycin, and 1 mg/ml neomycin) in drinking water for a total of 21 days, while the control group received drinking water only. During antibiotic treatment, water bottles from both groups were replaced every 2–3 days. Fresh fecal pellets were collected from every mouse (antibiotic treated and nonantibiotic treated) at the end of the antibiotic treatment and immediately placed in a sterile tube. Half of the fecal sample was transferred into a tared Eppendorf tube, weighed, and homogenized in sterile 1xPBS containing 20% glycerol for a final concentration of 10 mg/ml with the help of Eppendorf pestle, and afterward stored at −80°C. The remaining half was immediately frozen in dry ice and stored at −80°C for nucleic acid extraction and 16S rRNA amplicon sequencing analyses.

### BM transplantation

Mice got lethally irradiated (2 × 6 Gy) at an age of 7 wk with a YXLON Maxishot (YXLON International GmbH), with a 3-h break between each dose. In parallel, BM cells were isolated from femurs and tibias from 8-wk-old donors. Epiphyses were cut, and BM was flushed out with RPMI-1640 (Gibco) medium using a 1-ml syringe (with 27 G needles), filtered through a 70-µm strainer, counted, and resuspended in 100 μl 1xPBS for retro-orbital injection into recipient mice. 5 × 10^6^ BM cells were injected 4 h after the final irradiation, with mice maintained under light isoflurane anesthesia (2% isoflurane 2 liters/min O_2_). Mice were housed for 8 wk to allow for complete reconstitution before the start of the respective experiments.

### Colitis score—histological preparation and scoring

Pathological scoring was performed on day 10 after colitis induction by DSS. Colons (without cecum) were dissected, measured (length), cut open longitudinally, and rolled up starting from the distal colon. Colon rolls were fixed in 4% paraformaldehyde in 1xPBS for a minimum of 24 h. The samples were further paraffin embedded and sectioned at 5-μm thickness. Sections were stained with hematoxylin-eosin and scored for mild, moderate, or severe colitis according to the following parameters: crypt damage (0 = none, 1 = 1/3 loss of basal, 2 = 2/3 loss of basal, 3 = complete destruction), inflammatory cell infiltration (0 = none, 1 = mucosal, 2 = mucosal and submucosal/moderate, 3 = mucosal and submucosal/severe), epithelial erosion and ulceration (0 = none, 1 = light submucosal erosion, 2 = partial submucosal erosion, 3 = confluent submucosal erosion), and thickening (0 = none, 1 = mild/focal, 2 = partial, 3 = complete). Each score was multiplied by 0 (= 0%), 1 (= 1–25%), 2 (= 26–50%), or 3 (= 51–100%) depending on the % of the area involved. Scoring was performed blinded by a trained lab technician. Proximal, middle, and distal colon were first scored separately, and then a combined total score was calculated.

### Colon single-cell preparations for single-cell sequencing, flow cytometry, and FACS sorting

The large intestine was removed from freshly sacrificed animals, cut open longitudinally, and fecal contents were removed by vigorous shaking in PBS. For single-cell sequencing and FACS sorting of immune cells, colons were dissected as previously described (Campbell et al., 2020). Briefly, they were incubated at 37°C for 15 min in 25 ml IEL solution (2% FCS, 10 mM HEPES, 1% L-glutamine, 1% Pen/Strep, 1 mM DTT, and 1 mM EDTA in PBS) at 250 rpm. 1/10 of the cells dissociated using EDTA were directly lysed in RNA lysis buffer for the sequencing of ECs. Tissues were then briefly rinsed with PBS, transferred to 50-ml Falcons containing ceramic beads and 25 ml collagenase solution (2% FCS, 10 mM HEPES, 1% L-glutamine, 1% Pen/Strep, 1 U/ml DNAse I, and 1 mg/ml collagenase A in RPMI [Gibco]), and incubated for 30 min at 37°C rotating at 250 rpm. Released lamina propria contents were then filtered through a 100-μM cell strainer, and washed with 1xPBS containing 2% FCS to remove remaining collagenase. Colon endothelial cells of WT mice were isolated using another published protocol (Sokol et al., 2021), with some modifications: Before colon dissection, anesthetized mice were perfused with 4 ml of 1,000 U/ml heparin at a perfusion rate of 2 ml/min. The endothelial cell enrichment step using CD31 MicroBeads was skipped, and the obtained cells were directly stained as described below. For flow cytometry, colon single cells were incubated at 37°C for 20 min in 4 ml solution 1 (3% FCS, 20 mM HEPES, 5 mM EDTA, and 1 mM DTT in RPMI [Gibco]) at 180 rpm in a 6-well plate. Tissues were rinsed with 1xPBS, transferred to a fresh 6-well plate containing 4 ml of digest1 solution (0.8 mg/ml dispase, 20 mM HEPES, and 2 mM EDTA in RPMI), and again incubated at 37°C at 180 rpm for 5 min. Next, tissues were agitated vigorously in digest1 solution before transferring them into digest2 solution (0.1 mg/ml liberase TL, 0.5 mg/ml DNAseI, and 20 mM HEPES in RPMI). After another incubation for 20 min, tissue and solution were transferred into a gentleMACS M tube and dissociated in an octo-gentleMACS dissociator (Myltenyi Biotech). Suspensions were then filtered through a 100 μM cell strainer, and washed with 1xPBS containing 2% FCS to remove remaining enzymes.

### Flow cytometry and cell sorting

For flow cytometry, cells were resuspended in antibody mixtures (all from BioLegend: fixable viability dye eF780, CD16/CD32 Fc block, CD45 PE Texas Red/AF700/PerCP Cy5.5, CD3 FITC/PE Texas Red, CD19 FITC/BV605, F4/80 FITC/PB, CD11b AF700/PECy7, Ly-6G PECy-7/FITC, CD8 BV510, CD4 PerCP Cy5.5, NK1.1 APC, Ly-6C BV510, CD11c PE Texas Red, BST2 PE, B220 AF700, and CD103 APC) and stained for flow cytometry as previously described (Gawish et al., 2022) (Table 1). For single-cell sequencing, cells were resuspended in antibody mixtures, stained for 30 min in the dark, and viable CD45^+^ and CD45^−^ cells were sorted using a FACS ARIA into 1xPBS/0.2% BSA. Sorted cells were then pooled, counted, and fixed for 18 h at 4°C in 1 ml of freshly prepared fixation buffer (Fixation of Cells & Nuclei for Chromium Fixed RNA Profiling). On the next day, cells were pelleted, resuspended in freezing solution, and stored at −80°C until processing for fixed single-cell sequencing. Library preparation was performed using the Chromium mouse transcriptome probe set v1.0.1 protocol. Libraries were sequenced on an Illumina HiSeq 4000, and count matrices were generated using default parameters of the CellRanger version 7.1.0 software for the fixed assay. For the sequencing of immune cells, B cells (CD45^+^ and CD19^+^), T cells (CD45^+^, CD19^−^, and CD3^+^), neutrophils (CD45^+^, CD3^−^, CD19^−^, Ly-6G^+^, and CD11b^+^), and a myeloid cell–enriched fraction (CD45^+^, CD3^−^, CD19^−^, Ly-6G^−^, and CD11b^+^) were sorted directly into RNA lysis buffer. Endothelial cells and a fibroblast/SMC-enriched fraction were stained with anti-CD31, anti-EpCAM, and anti-CD45 antibodies and sorted into RNA lysis buffer (endothelial cells: CD45^−^, EpCam^−^, and CD31^+^; fibroblast/SMC fraction: CD45^−^, EpCam^−^, and CD31^−^).

### scRNA-seq analysis

Quality control was performed per sample on the pre-filtered CellRanger output matrices. Data were imported as Seurat (v.4.3.0) or Scanpy AnnData (v.1.9.1) objects and processed with custom R (v.4.1.0) or Python (v.3.8.12) scripts, respectively throughout the analysis.

Cells with mitochondrial reads >5%, hemoglobin reads >10%, or total unique molecular identifiers (UMI) count <1,000 were filtered out. Only genes with at least three reads in three cells were retained. Doublet detection was performed with the SOLO adaptation of single-cell variational inference (SCVI) and DoubletFinder using default settings. Cell barcodes identified by either method were labeled as doublet. In addition, we used the SCVI latent space to identify clusters that are significantly enriched with doublet cells. All cells and clusters identified as doublets were excluded. In a last step, we removed cells with gene counts in the lower 1% quantile. For integration, the samples were first merged before configuring an SCVI model with the sample name as a batch key and the genotype and treatment as categorical covariates. The model was trained with two hidden layers for the encoder, a negative binomial distribution for the gene likelihood (Luecken et al., 2022), and otherwise default settings. A neighborhood graph (n_neighbors = 50) was constructed on the latent space and used for Leiden clustering and uniform manifold approximation and projection embedding with Scanpy. Cell type annotation of the Leiden cluster was performed using marker gene expression and supported by SingleR annotation with the ImmGen reference. Clusters with ambiguous marker gene expressions were subclustered using resolution parameters ranging from 0.1 to 0.2. For the final annotation, clusters with the same cell type label were merged and evaluated by computing marker gene expression with the Seurat implementation of the Wilcoxon test. The cell type annotation was transferred to additional WT samples using SCANVI (Xu et al., 2021). To measure the genotype effect on baseline or DSS-treated cells, we performed differential gene expression analysis with MAST. The analysis was restricted to cell types with at least 10 cells per group. The differential expression analysis (DEA) results were ranked by the log_2fold change_ signed −log_10_ adjusted P value for gene set enrichment analysis (Korotkevich et al., 2021, *Preprint*) with the MSigDB HALLMARK database. Cell–cell interaction was inferred with CellChat v2 (Jin et al., 2021, 2025).

### Distal colon bulk sequencing transcriptomics

Mice were treated with DSS or plain drinking water for five or seven plus 3 days, sacrificed, and colons dissected and flushed with 1xPBS. The distal 1/3 of the colon was processed for RNA extraction. Tissue was homogenized using Circonia Beads (1 mm) and a FastPrep-24 homogenizer (VWR; Avantor). Total RNA was subjected to poly-A RNA enrichment (NEBNext Poly(A) mRNA Isolation Module; NEB) followed by stranded library preparation (NEBNext Ultra Directional RNA Library Prep Kit for Illumina; NEB), multiplexed, and sequenced on a NextSeq Illumina platform to a minimum of 30 Mio reads per sample. The 5-day time point used 75-bp single-end sequencing, and the 10-day time point used 100 bp of paired-end sequencing mode. Quality control and filtering of the raw reads was performed with FASTP. The reads were then mapped to the GRCm38 reference genome using the GENCODE M25 annotation with STAR (v.2.7.9a). The BAM files were converted to a unified count matrix with featureCounts. For DEA, the data were first subset for each comparison. Lowly expressed genes were then filtered out by only retaining genes with at least five counts in two samples in either group. The count matrix was then processed with the Limma “voomWithQualityWeights” function before applying the standard Limma workflow for DEA. The DEA results were ranked by the log_2fold change_ signed −log_10_ adjusted P value for gene set enrichment analysis (Korotkevich et al., 2021, *Preprint*) with the MSigDB HALLMARK database.

### Determination of FLNA Q/R site editing levels

For the determination of FLNA editing ratios in whole tissue samples, total RNA was isolated from homogenized human biopsies or mouse tissue with TRIfast reagent using the manufacturer’s protocol. After DNase I treatment, cDNAs were synthesized using LunaScript Reverse Transcriptase and random hexamer primers. An FLNA cDNA fragment spanning spliced exons 42–43 was amplified by PCR, gel-eluted, and Sanger sequenced to check editing levels (human and mouse primers used for amplification and sequencing; see supplementary material table). For human biopsies, two samples per location (2x proximal and 2x distal colon) were obtained and analyzed. In cases where the Sanger sequencing chromatogram was of inferior quality (background of all other bases >5–10%) in one of the samples, only the high-quality sample was used for statistical analysis. In cases where both proximal or both distal samples had a good and comparable quality of their Sanger sequencing chromatograms, their average was used to calculate editing frequencies.

For the detection of FLNA editing levels in sorted cell populations, RNA was extracted using the NucleoSpin 96 RNA Core Kit (Macherey-Nagel). Carrier RNA (Macherey-Nagel) was added to samples with cell counts <10^4^ cells. cDNA was synthesized using the LunaScript RT Mastermix (NEB) and then subjected to a two-step PCR: In the first round, Flna editing site primers (amplification of the region around the editing site) and Q5 High-Fidelity DNA Polymerase (NEB) were used to perform 25 cycles. 2 μl of this PCR served as a template for PCR2: Using the ampSeq barcoding primers for 15 cycles, thereby adding Illumina-compatible adapters with dual indices. PCR products were gel-eluted using the Monarch DNA Gel Extraction Kit (NEB) followed by quantification by Qubit Fluorometry (Thermo Fisher Scientific). A maximum of 96 samples were pooled into one multiplex for sequencing on an Illumina NovaSeq, or NextSeq instrument. After quality control using Fastqc and Multiqc, trimming was performed in two consecutive steps with Cutadapt ( --nextseq-trim = 30 --minimum-length = 10 (a) nextseq-trim; then: -a -A --minimum-length = 10), resulting in, on average, 58,000 processed reads/sample (150-bp paired-end). Reads were mapped using Hisat2, and editing ratios of each sample were calculated using Jacusa2.

### RT-quantitative PCR (qPCR)

RNA was isolated with NucleoSpin RNA Core Kit, including lyophilized carrier RNA for sorted cells (Macherey-Nagel). For real-time PCR assays, cDNA synthesis was performed using the LunaScript cDNA Synthesis Kit (NEB), according to manufacturer’s protocol. Real-time PCR was performed with SYBR Green Master Mix reagents (Avantor; VWR) on a StepOnePlus Real-Time PCR System (Applied Biosystems). Mouse primers were used for amplification of cytokines, *Flna*, *Adar*, and *Adarb1* are found in the Table S1. Transcript levels were normalized to *Gapdh*.

### Legendplex

Tissue cytokines and chemokines were measured using the LEGENDplex Mouse Anti-Virus Response Panel (13-plex) with V-Bottom Plate (BioLegend). Samples were prepared according to the manufacturer’s protocols and analyzed by flow cytometry. Data analysis was performed using the LEGENDplex data analysis software.

### Assessment of intestinal epithelial barrier permeability

To test the intestinal permeability in vivo, FLNA^Q^ and FLNA^R^ mice were starved for 4 h followed by oral gavage with 0.6 mg/g body weight FITC-dextran 4000. Serum was collected 4 h after gavage, and concentrations were measured by fluorometry (excitation: 485 nm, emission: 528 nm; EnSight; Perkin Elmer).

Barrier permeability was additionally assessed ex vivo. Colons of sacrificed FLNA^R^ and FLNA^Q^ mice were flushed with 1xPBS and filled with 1 ml FITC-dextran 4000 (1 mg/ml in PBS). Colons were closed from both sides and incubated at room temperature for 40 min. Following incubation, colons were fixed and prepared for paraffin sectioning. Proximal and distal colons were sectioned horizontally into two different segments each, and FITC-positive cells were visualized using fluorescence microscopy after DAPI staining in an Olympus VS120-S6 fluorescence slide scanning microscope.

### Fecal pellet output measurement

FLNA^Q^ and FLNA^R^ mice were kept separated by genotype in cages (four mice/cage) for 24 h with paper towel sheets instead of regular bedding. After 24 h sheets, were removed and air-dried for another 24 h, and the pellet number and dry weight were assessed.

### DNA isolation from fecal pellets and 16S rRNA gene amplification, sequencing, and analysis

DNA was isolated with the QIAamp Fast DNA Stool Kit according to the manufacturer’s instructions in an automated manner using a QiaCube Symphony at the Joint Microbiome Facility (Vienna, Austria). Amplification of bacterial and archaeal 16S rRNA genes from DNA samples was performed with a two-step barcoding approach. In the first-step PCR, the primers 515F and 806R, including a 5′-head sequence for two-step PCR barcoding, were used. PCRs, barcoding, library preparation, and Illumina MiSeq sequencing were performed by the Joint Microbiome Facility (Vienna, Austria) under project number JMF-2005-3. First-step PCRs were performed in triplicate (12.5 μl vol. per reaction) with the following conditions: 1XxDreamTaq Buffer (Thermo Fisher Scientific), 2 mM MgCl_2_ (Thermo Fisher Scientific), 0.2 mM dNTP mix (Thermo Fisher Scientific), 0.2 µM of forward and reverse primer each, 0.08 mg/ml BSA (Thermo Fisher Scientific), 0.02 U DreamTaq Polymerase (Thermo Fisher Scientific), and 0.5 μl of DNA template. Conditions for thermal cycling were 95°C for 3 min, followed by 30 cycles of 30 s at 95°C, 30 s at 52°C, and 50 s at 72°C, and finally 10 min at 72°C. Triplicates were combined for barcoding (8 cycles). Barcoded samples were purified and normalized over a SequalPrep Normalization Plate Kit using a Biomek NXP Span-8 pipetting robot (Beckman Coulter) and pooled and concentrated on columns (Analytik Jena). Indexed sequencing libraries were prepared with the Illumina TruSeq Nano Kit and sequenced in paired-end mode (2 × 300 bp; v3 chemistry) on an Illumina MiSeq following the manufacturer’s instructions. The workflow systematically included four negative controls (PCR blanks, i.e., PCR-grade water as template) for each 90 samples sequenced.

Amplicon pools were extracted from the raw sequencing data using the FASTQ workflow in BaseSpace (Illumina) with default parameters. Input data were filtered for PhiX contamination with BBDuk. Demultiplexing was performed with the Python package “demultiplex,” allowing one mismatch for barcodes and two mismatches for linkers and primers. Barcodes, linkers, and primers were trimmed off using BBDuk, with 47 and 48 bases being left-trimmed for F.1/R.2 and F.2/R.1, respectively. DADA2 R package was used for demultiplexing ASVs using a previously described standard protocol. FASTQ reads were trimmed at 150 nts with allowed expected errors of two. Taxonomy was assigned to 16S rRNA gene sequences based on SILVA taxonomy (release 138) using the DADA2 classifier. Amplicon sequence libraries were analyzed using the vegan (v2.4.3) and phyloseq (v1.30.0) packages of the software R. DESeq2 (v1.26.0) implemented in phyloseq was used to determine statistically significant differences in ASV abundances between groups of mice. Only ASVs that had ≥10 reads were considered for comparisons by DESeq2 analyses. All statistical analysis on microbiome data was carried out with the software R (R 4.0.2), and statistical tests and P values are indicated in the figure legends. The 16S rRNA gene sequences were deposited in the NCBI Sequence Read Archive (BioProject ID: PRJNA1051489).

### SCFA measurements

Fecal pellets (average weight 21.62 ± 4.90 mg) were suspended in 0.5 ml Milli-Q water (Milli-Q; Merck Millipore) and homogenized with a pellet mixer (VWR; Radnor). Homogeneous suspensions were briefly vortexed at maximum speed after adding 1 ml Milli-Q water. The supernatant after centrifugation samples for 5 min at 10,000 *g* was filtered over a 0.2-µm filter (0.2-µm sterile syringe filter, diameter 26 mm, Minisart, Sartorius, Göttingen, Germany). Filtered samples were analyzed on a 930 Compact IC Flex instrument equipped with an 858 Professional Sample Processor with extended MiPuT (Micro Metrohm Intelligent Partial Loop Injection Technique), a Metrohm CO_2_ Suppressor for inline bicarbonate removal, a Metrosep Organic Acids 250/7.8 column, a Metrosep Organic Acids Guard/4.6 guard column, and an 850 IC conductivity detector (Metrohm). Data were analyzed using the software R (R 4.0.2), and statistical tests and P values are indicated in the figure legends.

### Quantification of fecal bacterial density by flow cytometry

Microbial loads from mouse fecal samples preserved in 1xPBS containing 20% glycerol were determined using flow cytometry and counting beads as detailed below. Samples were diluted 10–500 times in 1xPBS. To remove any additional debris from the fecal matrix, samples were transferred into a flow cytometry tube by passing the sample through a snap cap containing a 35-μm pore size nylon mesh. Next, 500 μl of the microbial cell suspension was stained with SYTO 9 (0.5 μM in DMSO) for 15 min in the dark. The flow cytometry analysis of the microbial cells present in the suspension was performed using a BD FACS Melody (BD Biosciences), equipped with a BD FACSChorus software (BD). Briefly, background noise of the machine and of PBS was detected using the parameters forward scatter and side scatter. Microbial cells (no beads added) were then displayed using the same settings in a scatter plot using the forward scatter and side scatter and pre-gated. Singlets discrimination was performed. Absolute counting beads added to each sample were used to determine the number of cells per ml of fecal slurries by following the manufacturer’s instructions. Fluorescence events were monitored using the blue (488 nm—staining with SYTO 9 and CountBrightTM beads) and yellow-green (561 nm—CountBright beads only) optical lasers. The gated fluorescence events were evaluated on the forward–sideways density plot to exclude remaining background events and to obtain an accurate microbial cell count. Instrument and gating settings were identical for all samples (fixed staining–gating strategy).

### BM neutrophil isolation and BMDM differentiation

BM neutrophils were isolated from femurs of 8–10-wk-old mice using the Neutrophil Isolation Kit, Mouse (Miltenyi Biotec), following the provided protocol. In brief: femurs were flushed using a 25-gauge needle and BM cells were gently dissociated using a 1000-μl pipette tip and filtered through a 30-µm cell strainer. Cells were then sequentially incubated with the antibody cocktail and the microbeads provided in the kit, followed by negative selection on a QuadroMACS magnetic MACS separator using LS columns (both Miltenyi Biotec). For the preparation of BMDMs, BM was flushed from femurs with PBS, and cells were differentiated for 6 days in RPMI 1640 supplemented with 10% FCS, 1% pen-strep, and 10% L929 conditioned medium.

### Neutrophil DNA release assay

DNA release as a measure for neutrophil DNA trap release was performed as before (Ibrahim et al., 2024b). In short, 1 × 10^5^ BM neutrophils suspended in 100 μl of HBSS with Ca^2+^ and Mg^2+^ (HBSS^++^) were seeded and then stimulated with 0.5 μM of a23187 or with 0.1 nM of PMA for 300 min at 37°C. For quantification of DNA release, 5 μM of SYTOX Green was added, and fluorescence was measured at 15-min intervals at excitation/emission of 485/520 nm using the VarioSkan Lux plate reader (Thermo Fisher Scientific Inc).

### NETosis immunofluorescence

Colon sections (5 μm) were analyzed for neutrophils (Ly6G) and NETs (CitH4 and DNA) by immunofluorescence staining as reported in Ibrahim et al. (2024a). Sections were deparaffinized and rehydrated using xylene and decreasing concentrations of ethanol. After antigen retrieval in 0.5 M citrate buffer, colon sections were blocked in 1xPBS with 5% BSA and then stained for NETs with rat anti-mouse Ly6G for neutrophils (1:1,000), rabbit polyclonal anti-CitH4 antibody (1:500), and secondary antibodies: Cy3-conjugated donkey anti-rat IgG and Alexa Fluor 647–labeled donkey anti-rabbit IgG (1:400), as well as Hoechst 33342 (1:1,000) for the nuclear stain. Stained sections were imaged with an automated Axio Observer Z1 microscope (Carl Zeiss MicroImaging, Inc.) with a 20× objective and the TissueFAXS scan software. The tissue scoring of neutrophils and NETs was conducted manually by ordinal scoring based on four levels of signal frequency: 0–none, 1–few, 2–moderate, and 3–high number of detectable cells.

### CD8^+^ T cell depletion

8–10-wk-old male mice received either anti-mouse CD8α antibody (CD8 YTS169; 500 µg, i.p*.*) or the same volume of 1xPBS starting at 36 h before DSS treatment start and then again on days 1 and 3 after DSS start (with 2% DSS in drinking water ad libitum for 5 days). Mice underwent daily checkups, including weight measurements.

### Neutrophil migration speed measurements

To distinguish cells by genotype in a microfluidic device, FLNA^Q^ and FLNA^R^ BM neutrophils were counted and stained with 10 µM TAMRA and CFSE (Thermo Fisher Scientific) at 1,000 cells/μl for 10 mins at room temperature in the dark. Cells were washed three times and then mixed in equal numbers of FLNA^Q^ and FLNA^R^. To exclude dye effects on the cells, dye-swap controls were included in every experimental set.

Microfabricated polydimethylsiloxane (PDMS) devices were manufactured as described in Renkawitz et al. (2018). The pillar forest had pillars of 3.8-µm height and 10-µm width in a square layout. The pore size between pillars was 5 µm, and the analysis area was 0.7 × 2.5 mm in size. PDMS devices were preincubated with RPMI with 2% BSA prior to use and loaded on one side with 50 µM N-formylmethionine-leucyl-phenylalanine (fMLP) and on the other side with BMDNs (20,000 cell/μl). After 1 h of incubation, movies of stained cells in the pillar maze were acquired by time-lapse acquisition (90-s intervals) using a Nikon Ti2E inverted widefield microscope with a Plan Apo λ 20×/0.75 differential interference contrast 1 air perfect focus system objective and a NIKON FX-format, monochrome CMOS sensor camera. During imaging, devices were kept in a custom-built climate chamber (37°C, 5% CO_2_, and humidified). Spot analysis to calculate the mean cell speed was performed using the tracking tool of Imaris 9.9.1 software (Oxford Instruments). Only the first 40 frames (= 1-h observation time) were used for cell tracking.

### Statistical analysis

Data were analyzed using GraphPad Prism 9.1 (GraphPad Software). All details about statistical testing of individual experiments are explained in the respective legends. In brief, data are represented as a mean ± SD or SEM whenever indicated. Differences between two groups were tested using Student’s *t* test (two-sided) or nonparametric Mann–Whitney *U* test (for histological scores). For multiple group comparisons, data were analyzed using one-way ANOVA or two-way ANOVA (for weight-loss curves or editing time courses) or Kruskal–Wallis test (for histological scores), post hoc followed by a Tukey multiple comparison test. Statistical significance was attributed to P values ≤0.05. Measurements were taken from distinct samples (unless specified, e.g., distal and proximal colon), except for mouse weight measurements, which were taken from the same individuals on different days.

### Materials

The lists of reagents or resources are given in Table 1.

**Table 1.** Reagent or resource

Reagent or resource	Source	Identifier
Antibodies		
CD16/CD32 Fc block	eBioScience; Thermo Fisher Scientific	RRID:AB_467133
CD45 PE Texas Red/AF700/PerCP Cy5.5	Biolegend	RRID:AB_2564002/AB_493714/AB_893340
CD3 FITC/PE Texas Red	Biolegend	RRID:AB_312661/AB_2565882
CD19 FITC/BV605	Biolegend	RRID:AB_313640/AB_2564000
F4/80 FITC/PB	Biolegend	RRID:AB_893500/AB_893475
CD11b AF700/PECy7	Biolegend	RRID:AB_493705/AB_312798
Ly-6G PECy-7/FITC	Biolegend	RRID:AB_1877261/AB_1236488
CD8 BV510	Biolegend	RRID:AB_2563057
CD4 PerCP Cy5.5	Biolegend	RRID:AB_893324
NK1.1 APC	Biolegend	RRID:AB_313396
Ly-6C BV510	Biolegend	RRID:AB_2562351
CD11c PE Texas Red	Thermo Fisher Scientific	RRID:AB_10373971
BST2 PE	Biolegend	RRID:AB_1953283
B220 AF700	Biolegend	RRID:AB_493716
CD103 APC	Biolegend	RRID:AB_2563906
CD31 Pacific blue	Biolegend	RRID:AB_10613457
EpCam PE	Biolegend	RRID:AB_1134172
Ly6G 1A8	Biolegend	RRID:AB_1089180
CitH4 (citrulline 3)	Merck/MilliporeSigma	Cat#07–596
Cy3-conjugated donkey anti-rat IgG	Dianova	Cat#712-165–153
AF 647–labeled donkey anti-rabbit IgG	Thermo Fisher Scientific	RRID:AB_2536183
Anti-mouse CD8α, clone YTS169	In-house	NA
Biological samples		
Human sample 1	Proximal and distal colon biopsies of healthy patient	Ethics Committee number: 1692/2020
Human sample 2	Proximal and distal colon biopsies of healthy patient	Ethics Committee number: 1692/2020
Human sample 3	Proximal and distal colon biopsies of patient with active UC	Ethics Committee number: 1692/2020
Human sample 4	Proximal and distal colon biopsies of patient in remission from UC	Ethics Committee number: 1692/2020
Human sample 5	Proximal and distal colon biopsies of patient with active UC	Ethics Committee number: 1692/2020
Human sample 6	Proximal and distal colon biopsies of patient with active UC	Ethics Committee number: 1692/2020
Human sample 7	Proximal and distal colon biopsies of healthy patient	Ethics Committee number: 1692/2020
Human sample 8	Proximal and distal colon biopsies of healthy patient	Ethics Committee number: 1692/2020
Human sample 9	Proximal and distal colon biopsies of healthy patient	Ethics Committee number: 1692/2020
Human sample 10	Proximal and distal colon biopsies of patient in remission from UC	Ethics Committee number: 1692/2020
Human sample 11	Proximal and distal colon biopsies of patient in remission from UC	Ethics Committee number: 1692/2020
Human sample 12	Proximal and distal colon biopsies of healthy patient	Ethics Committee number: 1692/2020
Human sample 13	Proximal and distal colon biopsies of healthy patient	Ethics Committee number: 1692/2020
Human sample 14	Proximal and distal colon biopsies of healthy patient	Ethics Committee number: 1692/2020
Human sample 15	Proximal and distal colon biopsies of healthy patient	Ethics Committee number: 1692/2020
Human sample 16	Proximal and distal colon biopsies of patient in remission from UC	Ethics Committee number: 1692/2020
Human sample 17	Proximal and distal colon biopsies of patient with active UC	Ethics Committee number: 1692/2020
Human sample 18	Proximal and distal colon biopsies of patient with active UC	Ethics Committee number: 1692/2020
Human sample 19	Proximal and distal colon biopsies of patient in remission from UC	Ethics Committee number: 1692/2020
Human sample 20	Proximal and distal colon biopsies of patient in remission from UC	Ethics Committee number: 1692/2020
Human sample 21	Proximal and distal colon biopsies of patient with active UC	Ethics Committee number: 1692/2020
Chemicals, peptides, and recombinant proteins		
Dextran sulfate sodium salt, colitis grade (36,000–50,000)	MP Biomedicals	Cat#0216011080
Peqlab peqGOLD TriFast, DNA/RNA/protein purification reagent	Avantor, VWR Chemicals	Cat#30–2010
LunaScript Reverse Transcriptase (LunaScript RT Mastermix)	New England Biolabs GmbH	Cat#E3025
Fluorescein isothiocyanate–dextran, average mol weight 3,000–5,000	Merck/MilliporeSigma	Cat#FD4
Ampicillin sodium salt ≥97%, BioScience Grade	Carl Roth GmbH + Co	Cat#K029.1
Vancomycin hydrochloride	Carl Roth GmbH + C. KG	Cat#0242.3
Streptomycin sulfate	Carl Roth GmbH + Co	Cat#0236.1
Neomycin sulfate	Carl Roth GmbH + Co	Cat#8668.1
DreamTaq DNA Polymerase (5 U/μl)	Thermo Fisher Scientific	Cat#EP0705
SYTO 9 green fluorescent nucleic acid stain	Thermo Fisher Scientific	Cat#S34854
eBioscience fixable viability dye eFluor 780	Thermo Fisher Scientific	Cat#65–0865-14
CountBright absolute counting beads, for flow cytometry	Thermo Fisher Scientific	Cat#C36950
DNAse I	Merck/MilliporeSigma	Cat#11284932001
Collagenase A	Gibco; Thermo Fisher Scientific	Cat#17018029
Dispase II	Gibco; Thermo Fisher Scientific	Cat#17105041
Liberase TL TL Research Grade	Fisher Scientific; Thermo Fisher Scientific	Cat#NC1328423
Hoechst 33342	Thermo Fisher Scientific	Cat#62249
Citrate, 0.5 M buffer soln., pH 3.5	Thermo Fisher Scientific	Cat#15454239
SYTOX green	Thermo Fisher Scientific	Cat#S7020
a23187	Merck/MilliporeSigma	Cat#C7522
PMA	Merck/MilliporeSigma	Cat#19–144
N-Formyl-Met-Leu-Phe (fMLP)	Merck/MilliporeSigma	Cat#F3506
CellTrace CFSE cell proliferation kit	Thermo Fisher Scientific	Cat#C34570
Red-fluorescent tetramethylrhodamine (TAMRA) azide	Thermo Fisher Scientific	Cat#T10182
Q5 High-Fidelity DNA Polymerase	NEB	Cat#M0491
qScript cDNA synthesis-set	Avantor, VWR Chemicals	Cat#733–1175
Ultra-Plex 1-step ToughMix ROX (4X)	Avantor, VWR Chemicals	Cat#733–2466
PerfeCTa SYBR Green SuperMixes and FastMixes	Avantor, VWR Chemicals	Cat#733–1387
NucleoSpin 96 RNA Core Kit, 96-well Kit for RNA Isolation	Macherey-Nagel	Cat#740466.4
Lyophilized carrier RNA	Macherey-Nagel	Cat#740514
LunaScript RT SuperMix Kit	NEB	Cat#E3010
Monarch DNA Gel Extraction Kit	NEB	Cat#T1120
Critical commercial assays		
QIAamp Fast DNA Stool Kit	Qiagen	Cat#51604
SequalPrep normalization plate kit, 96-well	Thermo Fisher Scientific	Cat#A1051001
Illumina TruSeq Nano Kit	Illumina Inc.	Cat#A20015964
Fixation of Cells & Nuclei for Chromium Fixed RNA Profiling	10X Genomics,.Inc.	Cat#1000414
Chromium Fixed RNA Kit, Mouse Transcriptome, 4 rxns x 16 BC	10X Genomics, Inc.	Cat#PN-1000497
NEBNext Poly(A) mRNA Isolation Module	NEB	Cat#E7490
NEBNext Ultra Directional RNA Library Prep Kit for Illumina	NEB	Cat#E7765
LEGENDplex Mouse Anti-Virus Response Panel	Biolegend	Cat#740622
Neutrophil Isolation Kit, Mouse	Miltenyi Biotec	Cat#130-097-658
Deposited data		
Microbiome sequencing data	National Institutes of Health: BioProject - effect of filamin A editing on the gut microbiome	BioProject ID: PRJNA1051489
Bulk sequencing data of distal colon	National Institutes of Health: GEO dataset	GSE296059
scRNA-seq of colon cells	National Institutes of Health: GEO dataset	GSE296058
Flna amplicon sequencing (ampSeq)	National Institutes of Health: GEO dataset	GSE295910
Experimental models: Organisms/strains		
WT mice	C57BL/6J	RRID:IMSR_JAX:000664
FLNA^Q^ mice	Mouse breeding facility at Medical University of Vienna	Refs 8 + 9
FLNA^R^ mice	Mouse breeding facility at Medical University of Vienna	Refs 8 + 9
IL-10 KO mice	129(B6)-Il10tm1Cgn/J, the Jackson Laboratory	RRID:IMSR_JAX:004368
Vav1-Cre mice	Tg(Vav1-cre)1Cgp	MGI ID: 2450339
FLNA^QiR^ mice	Mouse breeding facility at Medical University of Vienna	This paper
LysM-Cre mice	B6.129P2-Lyz2^tm1(cre)Ifo^/J	RRID:IMSR_JAX:004781
Oligonucleotides (5′→3′)		
5′-GTC​AAG​TTC​AAC​GAG​GAG​CAC-3′	MicrosynthAG	Human Flna editing locus fw primer
5′-GTGCACCTTGG CATCAATTGC-3′	MicrosynthAG	Human Flna editing locus rev primer
5′-CCG​CCT​TAC​TGT​TTC​TAG​TCT-3′	MicrosynthAG	Mouse Flna editing locus fw primer
5′-GCT​GGT​TGA​CCT​TTA​ACC​CTG-3′	MicrosynthAG	Mouse Flna editing locus rev primer
5′-GTGYCAGCMGCCGCGGTAA-3′	MicrosynthAG	16S RNA barcoding 515F
5′-GGACTACNVGGGTWTCTAAT-3′	MicrosynthAG	16S RNA barcoding 806R
5′-ACA​CGA​CGC​TCT​TCC​GAT​CTG​AGC​ACA​TAC​CTG​ATA​GCC​CC-3′	MicrosynthAG	Flna editing detection fw primer for ampSeq
5′-CAG​ACG​TGT​GCT​CTT​CCG​ATC​TTC​TCT​CGT​GGG​ATG​AAA​CGC-3′	MicrosynthAG	Flna editing detection rev primer for ampSeq
5′-CAA​GCA​GAA​GAC​GGC​ATA​CGA​GAT​NNN​NNN​NNG​TGA​CTG​GAG​TTC​AGA​CGT​GTG​CTC​TTC​CGA​TCT-3′	MicrosynthAG	ampSeq—barcoding primers fw
5′-AAT​GAT​ACG​GCG​ACC​ACC​GAG​ATC​TAC​ACN​NNN​NNN​NAC​ACT​CTT​TCC​CTA​CAC​GAC​GCT​CTT​CCG​ATC​T-3′	MicrosynthAG	ampSeq—barcoding primers rev
5′-GAT​GAC​CAG​TCT​GGA​GGT​GC-3′	MicrosynthAG	Mouse Adar fw
5′-GCA​GCA​AAG​CCA​TGA​GAT​CG-3′	MicrosynthAG	Mouse Adar rev
5′-CAG​TTG​CAT​TTG​CCA​CAG​GTA​T-3′	MicrosynthAG	Mouse Adarb1 fw
5′-ACC​GTT​GAT​ACA​CTT​CGT​CCC-3′	MicrosynthAG	Mouse Adarb1 rev
5′-5′-GAA​CTG​GCA​GAA​GAG​GCA​CT-3′	MicrosynthAG	Mouse Tnf fw
5′-GGT​CTG​GGC​CAT​AGA​ACT​GA-3′	MicrosynthAG	Mouse Tnf rev
5′-CAA​AAT​ACC​TGT​GGC​CTT​GG-3′	MicrosynthAG	Mouse Il1b fw
5′-TAC​CAG​TTG​GGG​AAC​TCT​GC-3′	MicrosynthAG	Mouse IL1b rev
5′-CAG​CAA​CCA​TGG​GAG​AGA​ATG​CTG​A-3′	MicrosynthAG	Mouse Ifit1 fw
5′-GGC​ACA​GTT​GCC​CCA​GGT​CG-3′	MicrosynthAG	Mouse Ifit1 rev
5′-GAC​CAT​GGC​TGG​GAT​TCA​CC-3′	MicrosynthAG	Mouse Cxcl1 fw
5′-TCA​GAA​GCC​AGC​GTT​CAC​CA-3′	MicrosynthAG	Mouse Cxcl1 rev
5′-AAG​CTG​TAG​TTT​TTG​TCA​CCA​AG-3′	MicrosynthAG	Mouse Ccl2 fw
5′-CCA​TTT​GGT​TCC​GAT​CCA​GGT​TT-3′	MicrosynthAG	Mouse Ccl2 rev
5′-CCT​GGC​GGT​GCT​GAG​CTA​CT-3′	MicrosynthAG	Mouse Ifna fw
5′-TTC​TCC​TGC​GGG​AAT​CCA​AA-3′	MicrosynthAG	Mouse Ifna rev
5′-TGA​GGC​GCT​GTC​ATC​GAT​TT-3′	MicrosynthAG	Mouse Il10 fw
5′-CAT​GGC​CTT​GTA​GAC​ACC​TT-3′	MicrosynthAG	Mouse Il10 rev
5′-CCA​CGG​CCT​TCC​CTA​CTT​CA-3′	MicrosynthAG	Mouse Il6 fw
5′-TGC​AAG​TGC​ATC​GTT​GTT​C-3′	MicrosynthAG	Mouse Il6 rev
Software and algorithms		
BBDuk (BBTools)	Bushnell, B., https://sourceforge.net/projects/bbmap	NA
Python package demultiplex	Laros, J.F.J., https://github.com/jfjlaros/demultiplex	NA
DADA2 (v1.16.0) R package	https://www.r-project.org/, R 4.0.2	NA
Vegan (v2.4.3) R package	https://www.r-project.org/, R 3.4.0	NA
Phyloseq (v1.30.0) R package	https://www.r-project.org/, R 3.4.1	NA
DESeq2 (v1.26.0) R package	https://www.r-project.org/, R 3.4.1	NA
CellRanger (v7.1.0)	10X Genomics, Inc.	NA
Seurat (v.4.3.0)	https://github.com/satijalab/seurat/releases	NA
Scanpy AnnData (v.1.9.1)	https://scanpy.readthedocs.io/en/stable/installation.html	NA
SOLO (adaption of SCVI)	https://github.com/calico/solo	NA
DoubletFinder	https://github.com/chris-mcginnis-ucsf/DoubletFinder	NA
SCVI	https://github.com/YosefLab/scVI	NA
SingleR	https://github.com/dviraran/SingleR	NA
Scanpy	https://github.com/theislab/Scanpy	NA
MAST	https://www.bioconductor.org/packages/release/bioc/html/MAST.html	NA
FASTP	https://github.com/OpenGene/fastp	NA
STAR (v.2.7.9a)	https://github.com/alexdobin/STAR	NA
FeatureCounts	https://subread.sourceforge.net/featureCounts.html	NA
Limma	https://bioconductor.org/packages/release/bioc/html/limma.html	NA
R (v.4.1.0 or R 4.0.2) and python (v.3.8.12)	https://www.r-project.org/and https://www.python.org/downloads/release/python-3812/	NA
TissueFAXS scan software	TissueGnostics GmbH	NA
Imaris 9.9.1	Oxford Instruments	NA
Cutadapt	https://cutadapt.readthedocs.io/en/stable/installation.html	NA
Hisat2	https://daehwankimlab.github.io/hisat2/	NA
Jacusa2	https://github.com/dieterich-lab/JACUSA2	NA

### Online supplemental material

Fig. S1 shows immune cell composition of heathy mice, gating scheme of sc-preparations, and volcano plots of DEGs in naïve colon ECs. Fig. S2 shows the impact of the FLNA editing status in an IL-10 KO background; colon length shortening and scRNA clustering at day five of DSS; total numbers and unchanged immune cell populations after DSS treatment; gating strategy for FACS analysis of immune cell populations in colon. Fig. S3 shows correlation of a bacterial ASV with butyrate levels and colitis score and the validation of antibiotic microbiota depletion. Fig. S4 shows the genomic scheme of inducible FLNA^QiR^ mouse; body weight loss (BWL) curves of FLNA^QiR^ and Vav-Cre FLNA^QiR^, repopulation experiments after bone marrow transplantation (BMT); BWL and total histology score of the BMT experiment; BWL of Lysm-Cre FLNA^QiR^ and CD8^+^ T cell depletion experiment. Fig. S5 shows the correlation graphs of FLNA editing vs gene expression levels; expression of *Adar* and *Adarb1* and *Flna* from mutant and WT scRNA-seq data; images of NETosis in colons; inferred cell–cell interactions of monocytes/macrophages and neutros. Table S1 shows the cell numbers from scRNA-seq of naive mice. Table S2 shows the DEGs of scRNA-seq in naive mice. Table S3 shows the bulk RNA-seq of naive mice. Table S4 shows the bulk RNA sequencing results of colons of DSS treated mice. Table S5 shows the cell numbers from scRNA-seq of colons of DSS-treated mice. Table S6 shows the DEGs from scRNA-seq of colons of DSS-treated mice. Table S7 shows the clinical data.

## Supplementary Material

Table S1 shows the cell numbers from scRNA-seq of naive mice.

Table S2 shows the DEGs of scRNA-seq in naive mice.

Table S3 shows the bulk RNA-seq of naive mice.

Table S4 shows the bulk RNA sequencing results of colons of DSS treated mice.

Table S5 shows the cell numbers from scRNA-seq of colons of DSS-treated mice.

Table S6 shows the DEGs from scRNA-seq of colons of DSS-treated mice.

Table S7 shows the clinical data.

## Data Availability

Data are available in the article itself and its supplementary materials. Transcriptomic and amplicon sequencing data are available under GEO accession numbers GSE296058, GSE296059, and GSE295910. 16S rRNA gene sequences were deposited in the NCBI (https://www.ncbi.nlm.nih.gov/) Sequence Read Archive under BioProject ID PRJNA1051489.
